# ERCC6L-mediated stabilization of HIF-1α enhances glycolysis and stemness properties of lung adenocarcinoma cells

**DOI:** 10.1038/s41419-025-07879-4

**Published:** 2025-07-21

**Authors:** Zhimin Lu, Xuming Yao, Jialu Jiang, Guoxin Hou

**Affiliations:** 1 Department of Outpatient, Affiliated Hospital of Jiaxing University, The First Hospital of Jiaxing, Jiaxing, Zhejiang China; 2 Department of Oncology, Affiliated Hospital of Jiaxing University, The First Hospital of Jiaxing, Jiaxing, Zhejiang China

**Keywords:** Cancer stem cells, Cancer metabolism

## Abstract

Molecular signatures are increasingly recognized as pivotal factors in therapy selection for lung adenocarcinomas (LUAD). A developing therapeutic approach focuses on targeting metabolic dependencies within cancer cells. ERCC6L, a vital protein involved in chromosome separation during cell mitosis, emerges as a compelling subject concerning its impact on LUAD tumorigenesis and progression. Our investigation uncovered elevated levels of ERCC6L in LUAD, significantly associated with unfavorable patient outcomes. Functional analyses elucidate ERCC6L’s role in promoting LUAD cell proliferation, migration, and invasion by inducing glycolysis and stemness characteristics. Mechanistically, our data reveal ERCC6L’s involvement in upregulating aerobic glycolysis through the induction of hypoxia-inducible factor-1α (HIF-1α) expression and transcriptional activity in LUAD cells. Furthermore, ERCC6L stabilizes HIF-1α by inhibiting its hydroxylation and ubiquitin-mediated degradation. The ERCC6L/HIF-1α axis plays a crucial functional role in enhancing cancer stemness and LUAD progression both in vitro and in vivo. Hence, our findings underscore the significance of the ERCC6L/HIF-1α axis in regulating aerobic glycolysis in LUAD cells, suggesting its potential as a biomarker and therapeutic target for LUAD patients.

## Introduction

Lung cancer is the leading cause of cancer-related deaths globally [1], with non-small cell lung cancer (NSCLC) predominating as the primary histopathology subtype [2]. Within NSCLC cases, lung adenocarcinoma (LUAD) accounts for approximately 60% [3]. The prognosis for LUAD patients, particularly those in advanced stages, is unfavorable due to ineffective treatment methods and drug resistance, contributing significantly to tumor progression and poor patient outcomes. Hence, understanding the mechanisms propelling LUAD progression is vital for identifying potential therapeutic targets.

ERCC Exception Repair 6 Like (ERCC6L), a member of the Switch/Sucrose Non-Fermentable (SWI/SNF2) protein family, also known as PLK1-interacting checkpoint helicase (PICH), plays a crucial role in cell mitosis as a DNA helicase [4]. It is pivotal in the spindle assembly checkpoint, ensuring proper chromosome separation during mitosis. ERCC6L, acting as a DNA transposase, regulates spindle assembly checkpoint signals by relocating to endocentric and kinetochore regions of mitotic chromosomes [5]. It facilitates sister chromatid separation [6, 7], supporting cell survival and proliferation. Studies indicate that ERCC6L deletion leads to chromosomal abnormalities, embryonic lethality, DNA damage, p53 activation, and increased apoptosis, suggesting its potential as a therapeutic target [8, 9]. Aberrant expression of ERCC6L has been observed in various tumors, influencing processes such as cell proliferation, migration, and invasion [10–13]. While existing research suggests ERCC6L’s involvement in LUAD [14], the specific functional pathways and molecular mechanisms remain incompletely understood.

Cancer’s metabolic reprogramming, notably identified by Otto Warburg’s observations on increased glucose uptake and lactate production in cancer cells even in oxygen-rich environments (termed aerobic glycolysis), stands as a hallmark [15]. This distinct glycolytic shift, diverging from normal cells, underscores its significance in aggressive cancer behavior [16]. Targeting this aberrant metabolism presents a promising avenue for cancer therapy, evidenced by potent antitumor effects of glycolytic inhibitors like 2-deoxyglucose (2-DG) and 3-bromopyruvate (3-BrPA) [17]. At the core of drug resistance and metastasis lie cancer stem cells (CSCs), which notably favor glycolytic phenotypes [18]. This observation underscores the potential of targeting CSC metabolism in cancer therapy. Thus, delving into the key targets that modulate aerobic glycolysis in CSCs holds substantial value, despite the limited understanding of CSCs’ metabolic characteristics and regulatory mechanisms.

Elevated levels of hypoxia-inducible factor-1α (HIF-1α) are associated with advanced cancer progression and unfavorable clinical outcomes in LUAD patients [19]. HIF-1 pathway activation triggers metabolic reprogramming and enhances angiogenesis crucial for cancer advancement [20, 21]. Newly synthesized HIF-1α is swiftly hydroxylated at certain proline sites by oxygen-reliant dioxygenases known as PHDs, which leads to its ubiquitination and subsequent degradation, ultimately decreasing its protein stability [22–24]. The regulation of proline hydroxylation on HIF-1α, influenced by oxygen availability, is critical, with LUAD often exhibiting hyperactive HIF-1 pathways [25, 26]. However, the precise molecular mechanisms driving HIF-1 activation in LUAD are not fully understood.

Our study unveils a novel mechanism by which ERCC6L activation fosters glycolysis by stabilizing HIF-1α through hindering its hydroxylation and ubiquitin-mediated degradation. Targeting interventions toward ERCC6L could be beneficial in thwarting the normoxic HIF-1α signaling pathway.

## Methods and materials

### Cell lines and culture conditions

LUAD cell lines A549, H23, H1299, H1975, PC-9, and SPC-A1, as well as human bronchial epithelial (16HBE) cells, were obtained from the Shanghai Cell Bank of the Chinese Academy of Sciences. A549 and SPC-A1 cells were cultured in Dulbecco’s Modified Eagle’s Medium (DMEM; Gibco, Grand Island, NY, USA) supplemented with 10% fetal bovine serum (FBS; Gibco, Grand Island, NY, USA) and 1% penicillin/streptomycin (Invitrogen, Carlsbad, CA, USA). H23, H1299, H1975, PC-9, and 16HBE cells were maintained in RPMI-1640 medium (Gibco, Grand Island, NY, USA) supplemented similarly. All cells were incubated in a humidified incubator (Thermo Scientific, Waltham, MA, USA) at 37 °C with 5% CO₂. Hypoxic conditions were established by placing the cells in a modular hypoxia chamber (BioSpherix, Parish, NY, USA) flushed with a gas mixture containing 1% O₂, 5% CO₂, and 94% N₂.

### Tissue microarray and immunohistochemistry (IHC)

A lung adenocarcinoma tissue microarray (Cat# HLugA180Su06) was obtained from Outdo Biotech (Shanghai, China) and comprised 95 paired tumor and adjacent normal tissue samples. The clinical cohort included 41 females and 54 males, aged 20–84 years. Immunohistochemical (IHC) staining was quantified by calculating a composite score derived from two parameters: the percentage of positively stained cells and the staining intensity. For the positive area, scores were assigned as follows: <5% (score 0), 5–25% (score 1), 26–50% (score 2), 51–75% (score 3), and >75% (score 4). Staining intensity was rated as 0 (negative), 1 (weak), 2 (moderate), or 3 (strong). The final IHC score was determined by multiplying these two values, and a cutoff score of 6 was used to differentiate between high and low ERCC6L expression. Two experienced pathologists independently performed the assessments to ensure the accuracy and reproducibility of the results.

### RNA extraction and quantitative real-time PCR

Total RNA was extracted using TRIzol (Sigma-Aldrich, St. Louis, MO, USA) according to the manufacturer’s protocol. Reverse transcription was performed using RNAiso Plus reagent (TaKaRa, Japan). Quantitative real-time PCR (qRT-PCR) was carried out using SYBR Premix Ex Taq II (TaKaRa, Japan) on an ABI 7500 Real-Time PCR System (Applied Biosystems, Foster City, CA, USA). Relative gene expression was calculated using the 2^−ΔΔCt^ method with GAPDH as the internal control. Each experiment was independently performed in triplicate. Primer sequences are listed in Table 1.

**Table 1:** Primers used for qPCR assay.

Gene	Forward (5´–3´)	Reverse (5´–3´)
ERCC6L	CTCTGGCTTGCTACTTTATCGAG	TGCATCAAACATACCGGAAAGG
HIF1α	GAACGTCGAAAAGAAAAGTCTCG	CCTTATCAAGATGCGAACTCACA
OCT4	GTGTTCAGCCAAAAGACCATCT	GGCCTGCATGAGGGTTTCT
SOX2	GCCGAGTGGAAACTTTTGTCG	GGCAGCGTGTACTTATCCTTCT
NANOG	TTTGTGGGCCTGAAGAAAACT	AGGGCTGTCCTGAATAAGCAG
GLUT1	CTTTGTGGCCTTCTTTGAAGT	CCACACAGTTGCTCCACAT
HK-2	GATTGTCCGTAACATTCTCATCGA	CTTGCAGCAGGGCCAGGCAGTCAC
PDK1	CTGTGATACGGATCAGAAACCG	TCCACCAAACAATAAAGAGTGCT
LDHA	TGGAGATTCCAGTGTGCCTGTATGG	CACCTCATAAGCACTCTCAACCACC
PFKFB3	TTGGCGTCCCCACAAAAGT	AGTTGTAGGAGCTGTACTGCTT
PFKL	GCTGGGCGGCACTATCATT	TCAGGTGCGAGTAGGTCCG
PGK1	TGGACGTTAAAGGGAAGCGG	GCTCATAAGGACTACCGACTTGG
GAPDH	GGAGCGAGATCCCTCCAAAAT	GGCTGTTGTCATACTTCTCATGG

### CCK-8 assay

Cell proliferation was determined using the Cell Counting Kit-8 (CCK-8; Dojindo, Kumamoto, Japan) following the manufacturer’s instructions. Cells were seeded in a 96-well plate, treated accordingly, and incubated with 10 µL of CCK-8 solution at 37 °C for 1.5 h. Absorbance at 450 nm was measured using a BioTek ELx808 microplate reader (BioTek Instruments, Winooski, VT, USA).

### EdU Labeling and Immunofluorescence

Cells were seeded in 24-well plates and incubated with 50 mM 5-ethynyl-2’-deoxyuridine (EdU; RIBOBIO, Guangzhou, China) for 2 h, followed by staining with Apollo®567 as per the manufacturer’s protocol. Images were acquired using a Zeiss Axio Observer microscope (Carl Zeiss, Oberkochen, Germany), and the percentage of EdU-positive cells was quantified.

### Transwell migration and invasion assays

Transwell chambers (24-well; Corning, Corning, NY, USA) were used to assess cell migration and invasion. For the invasion assay, the upper chamber was pre-coated with Matrigel (BD Biosciences, San Jose, CA, USA). For both assays, 5 × 10^4^ cells suspended in serum-free medium were seeded in the upper chamber, while the lower chamber contained medium with 20% FBS. After 48 h, cells were fixed and stained with 0.1% crystal violet (Beyotime, Shanghai, China) for 20 min, washed with PBS, and counted using ImageJ software (National Institutes of Health, USA).

### Tumorsphere formation assay

A total of 20,000 cells were seeded in ultra-low attachment 6-well plates (Corning, Corning, NY, USA) and cultured in DMEM/F12 medium supplemented with 5 µg/mL insulin (Sigma-Aldrich, Taufkirchen, Germany), 2% B27 (Gibco, Grand Island, NY, USA), 20 ng/mL epidermal growth factor (R&D Systems, Minneapolis, MN, USA), 100 U/mL penicillin, and 100 µg/mL streptomycin (GIBCO, Rockville, MD, USA) for 7 days. Tumorspheres were defined as spheres with a diameter greater than 50 µm and quantified accordingly.

### Extracellular acidification rate (ECAR) analysis

ECAR was measured using the Agilent Seahorse XF24 Extracellular Flux Analyzer (Agilent Technologies, Santa Clara, CA, USA). Cells (1 × 10^5^ per well) were seeded in XF24 plates (Agilent Technologies, Santa Clara, CA, USA) and incubated overnight. Following washing with Agilent Seahorse XF buffer, sequential injections of glucose, oligomycin, and 2-deoxy-glucose were performed. ECAR values were normalized to cell number and expressed as mean ± SEM.

### Luciferase reporter assay

A 3× hypoxia response element (HRE; sequence: GCATACGTGGGCGCATACGTGGGCGC-ATACGTGGGC) was cloned into the PGL-3 Basic luciferase reporter plasmid (Promega, Madison, WI, USA), creating the HRE luciferase reporter. Cells were transiently co-transfected with the HRE reporter and the pRL-TK Renilla luciferase plasmid (Promega, Madison, WI, USA). Luciferase activity was measured 48 h post-transfection using the Dual-Luciferase Reporter Assay System (Promega, Madison, WI, USA), and data were normalized to Renilla luciferase activity.

### Establishment of stable cell lines

Lentiviral vectors for ERCC6L overexpression (Gene ID: 54821) with a C-terminal FLAG tag were obtained from General Biosystems (Anhui) Co., Ltd., while lentiviral particles for ERCC6L knockdown were packaged and purchased from Biosmedi (Shanghai, China). The targeting sequences for ERCC6L were 5′-TGCTCATTACCTAAGATATGTGA-3′ and 5′-TGGAAGAAAAGGTGGTATATTGG-3′. Exponentially growing cells were infected in six-well plates and selected with puromycin (Sigma-Aldrich, St. Louis, MO, USA). Knockdown or overexpression efficacy was confirmed by Western blot.

### Cell transfection and RNA interference

LUAD cells were transfected using Lipofectamine 3000 (Invitrogen, Carlsbad, CA, USA) according to the manufacturer’s protocol. For HIF1α knockdown, siRNAs (synthesized by GenePharma, Shanghai, China) were used. The siRNA targeting HIF1α had the sequence: sense 5′-CCACAGACAUCAAGGGCAA-3′, with a non-specific control (si-NC) sequence: sense 5′-UUCUCCGAACGUGUCACGUdTdT-3′.

### Western Blotting

Cells were lysed in RIPA buffer (KeyGEN, Nanjing, China) supplemented with protease inhibitors (PMSF; KeyGEN). Protein concentrations were determined using a BCA Kit (KeyGEN). Thirty micrograms of protein were separated by SDS-PAGE and transferred onto PVDF membranes. Membranes were blocked with 5% non-fat milk and incubated with primary antibodies, including anti-ERCC6L (15688-1-AP, Proteintech), anti-OCT4 (60242-1-Ig, Proteintech), anti-NANOG (14295-1-AP, Proteintech), anti-HIF1α (80933-1-PBS, Proteintech), anti-VHL (#77591, Cell Signaling Technology), and anti-GAPDH (#2118S, Cell Signaling Technology). Secondary antibodies (HRP-conjugated goat anti-rabbit IgG, Cell Signaling Technology, Danvers, MA, USA) were applied, and protein bands were visualized using enhanced chemiluminescence (ECL; Thermo Fisher Scientific, Waltham, MA, USA).

### Cytotoxicity assay for cisplatin

Cisplatin (Sigma-Aldrich, St. Louis, MO, USA) cytotoxicity was assessed by treating cells with varying concentrations of the drug for 72 h. Cell viability was then measured using the CCK-8 assay, and the IC50 was determined based on the dose–response curve.

### Apoptosis analysis

Cell apoptosis was evaluated using the Annexin V-FITC Apoptosis Detection Kit (Vazyme, #A211-02) according to the manufacturer’s instructions. Stained cells were analyzed using a FACScan flow cytometer (Millipore, Billerica, MA, USA) and data processed with FlowJo v7.6 software.

### Ubiquitination assay

Cells were co-transfected with HA-tagged ubiquitin and Flag-tagged HIF-1α plasmids. After 48 hours, cells were treated with MG132 (10 µM for 4 h; Sigma-Aldrich) and lysed in buffer containing 150 mM NaCl, 10 mM Tris, and 2% SDS. Lysates were precleared with Protein A/G beads (Santa Cruz Biotechnology, Dallas, TX, USA) for 1 h, then incubated with 1 µg anti-Flag antibody overnight at 4 °C. Following washing with PBS, precipitated proteins were analyzed by Western blot using an anti-HA antibody.

### Animal experiments

For in vivo studies, 5 × 10^6^ LUAD cells were subcutaneously injected into the right flank of 4-week-old male BALB/c nude mice (SPF, Beijing Biotechnology Co., Ltd., Beijing, China). Tumor growth was monitored, and mice were euthanized after 30 days for tumor excision, photography, and weighing. For the tail vein metastasis model, 1 × 10^6^ cells were injected per mouse. Lung metastases were evaluated by hematoxylin and eosin (HE) staining. All animal experiments were approved by Laboratory Animal Ethics Committee of the Medical College of Jiaxing University (JUMC2022-010).

### Proximity ligation assay (PLA)

PLA was conducted with the Duolink® in situ kit (DUO92101, Sigma–Aldrich). Cells grown on sterile glass coverslips were washed with PBS, fixed in 4% paraformaldehyde (20 min), and permeabilized with 0.1% Triton X-100 (5 min, 4 °C). Subsequent steps followed the manufacturer’s protocol, using primary antibodies diluted to 1:100. Imaging was performed on a ZEISS LSM880 microscope (Germany).

### Statistical analysis

Quantitative data are expressed as the mean ± standard deviation (SD). Normality of data distribution was assessed using the Shapiro–Wilk test. For comparisons between two groups, if data were not normally distributed, the non-parametric Mann–Whitney *U* test was applied; otherwise, an unpaired *t*-test was used. For comparisons among three or more groups, one-way ANOVA followed by Tukey’s post hoc test was employed for normally distributed data with equal variances, while the Kruskal–Wallis test with Dunn’s post hoc test was used for non-normally distributed data. Statistical analyses were performed using GraphPad Prism 9.0 software (GraphPad Software, Inc., San Diego, CA, USA), with a significance threshold set at *p* < 0.05.

## Results

### ERCC6L exhibited heightened expression in LUAD and correlated with an unfavorable prognosis

**Fig. 1:**
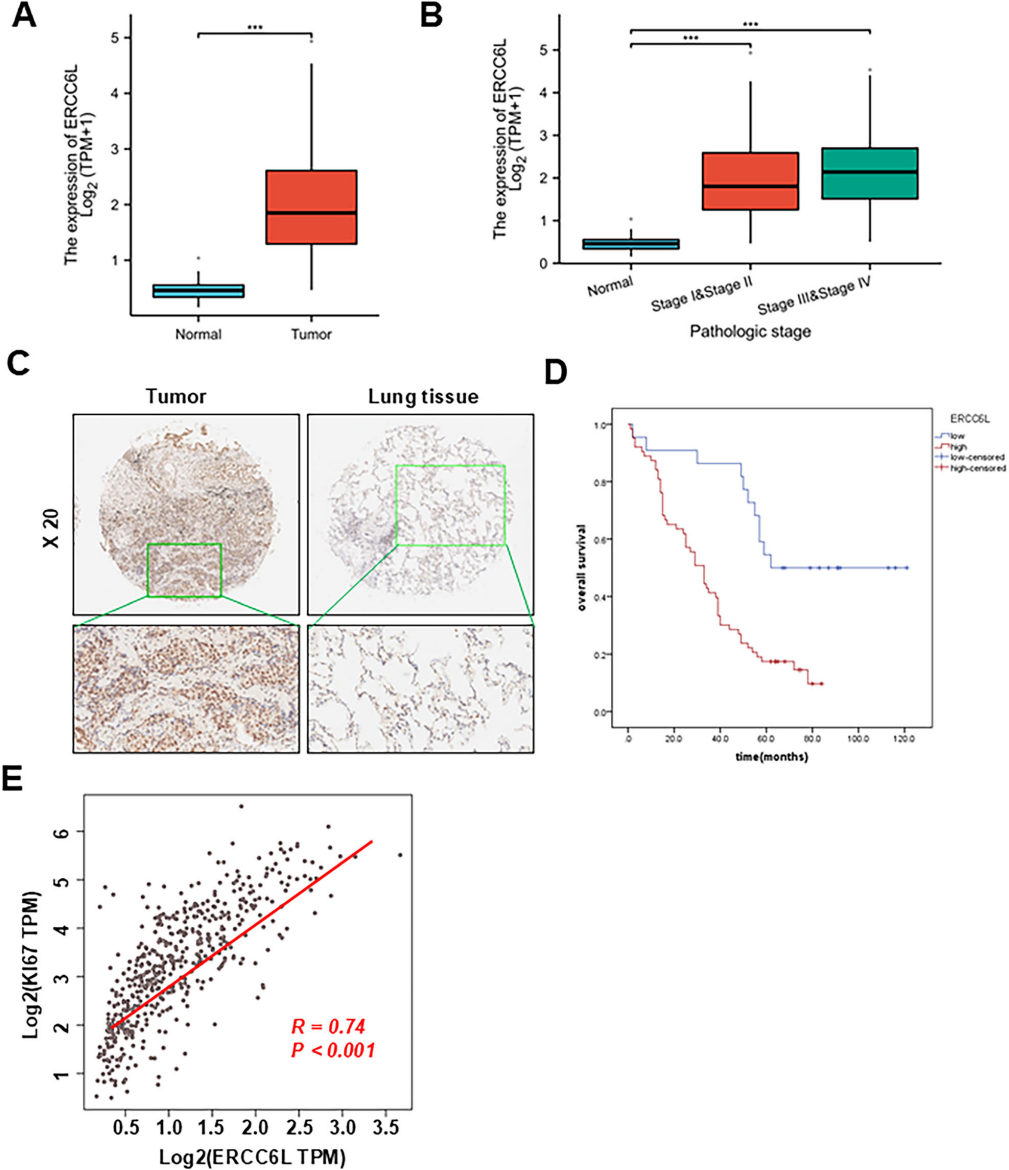
ERCC6L is elevated in human LUAD and correlates with an unfavorable prognosis. **A** ERCC6L expression in LUAD tissues versus adjacent normal tissues from the TCGA cohort (Normal, *n* = 59; Tumor, *n* = 515). **B** Association between ERCC6L expression and LUAD tumor stage from the UALCAN database. **C** Immunohistochemical detection of ERCC6L in an 85-paired LUAD TMA cohort. Representative images of non-tumor and tumor tissues are presented. **D** Kaplan–Meier analysis showing overall survival (HR = 0.74, *p* < 0.001). **E** Spearman correlation analysis of ERCC6L and Ki-67 mRNA levels in the TCGA database. Data are shown as the mean ± SEM. *p < 0.05, **p < 0.01, ***p < 0.001.

Analysis from the UALCAN database [27] indicated a marked upregulation of ERCC6L expression in LUAD tumors compared to normal lung tissue (Fig. 1A). Subsequently, we explored the potential association between ERCC6L expression in LUAD tissues and clinicopathological characteristics of patients in the TCGA dataset. As illustrated in Fig. 1B, increased ERCC6L expression showed a significant correlation with TNM stage (*p* < 0.05). Protein levels of ERCC6L were assessed in 85 LUAD tissue pairs via IHC, revealing significantly higher levels in tumor tissues compared to peritumoral lung tissues (Fig. 1C). Among 85 pairs of clinical samples, 61 out of 85 (71.8%) cancer tissues displayed increased ERCC6L expression compared to corresponding adjacent non-cancerous tissues (Table 2). Furthermore, based on cancer tissue staining intensity, we categorized 85 patients with available clinical data into high ERCC6L (*n* = 61) and low ERCC6L (*n* = 24) groups, and subsequently conducted statistical comparisons of various clinicopathological features between the two groups (Table 3). We also performed Cox proportional hazards regression analysis to exclude the confounder effect. Multivariate analysis confirmed that low ERCC6L expression was an independent predictor for reduced tumour-free survival of LUAD patients (Table 4). Our findings revealed a positive correlation between ERCC6L expression and TNM stages. Notably, Kaplan-Meier analysis of overall survival indicated that patients with elevated ERCC6L expression had significantly shorter overall survival times compared to those with lower ERCC6L expression (Fig. 1D), suggesting a strong association between high ERCC6L expression and unfavorable outcomes in LUAD patients. These results were consistent with the overall survival and disease-free survival analysis from our previous study [28]. Spearman correlation analysis indicated a positive correlation between ERCC6L and Ki-67 mRNA expression levels, suggesting a potential role of ERCC6L in promoting cell proliferation in LUAD (Fig. 1E). Additionally, ERCC6L expression was assessed in LUAD cell lines and human bronchial epithelial cells (HBE), revealing upregulation, particularly in A549 and SPC-A1 (Fig. S1A, B). These findings suggest an oncogenic potential for ERCC6L in LUAD.

**Table 2:** Differential expression of ERCC6L in cancerous and lung tissues.

	*n*	ERCC6L expression		Chi-square value	*p* Value
		High (%)	Low (%)		
Lung carcinoma	85	61	24	91.398	0.0001
lung tissues	85	1	84

**Table 3:** Correlation between ERCC6L expression and clinicopathological characteristics.

	variables	ERCC6L expression		Total	*χ*2	*p* Value
		Low	High			
Age (year)					2.088	0.149
	≤60	16	27	43		
	å 60	12	39	51		
T stage					1.235	0.266
	T1/T2	23	47	70		
	T3/T4	5	19	24		
Sex					1.013	0.314
	Female	10	31	41		
	male	18	35	53		
TNM stage					5.635	0.018
	Ι/II	22	34	56		
	III/IV	6	31	37		
	null					
N stage					1.582	0.209
	N0	17	25	42		
	N1/N2/N3	10	27	37		
	null					
M					0.435	0.509
	M0	28	64	92		
	M1	0	1	1		
	null					
VEGF					0.245	0.621
	Negative	13	27	40		
	positive	15	39	54		
	null					
Grade					0.001	0.973
	I/II	19	48	67		
	III	7	18	25		
	null

**Table 4:** Univariate and multivariate analyses of the factors correlated with the overall survival of lung carcinoma patients.

variables	Univariate analysis			Multivariate analysis		
	HR	95% CI	*p* Value	HR	95% CI	*p* Value
Expression	3.351	1.730–6.490	0.000	2.680	1.284–5.594	0.009
Sex	1.318	0.807–2.152	0.270			
Grade	1.908	1.196–3.044	0.007	2.104	1.233–3.593	0.006
Age	0.953	0.582–1.562	0.849			
T stage	1.446	1.059–1.974	0.020	1.034	0.674–1.586	0.879
N stage	1.732	1.325–2.264	0.000	1.392	0.996–1.943	0.053
M stage	1.099	0.152–7.964	0.926			
TNM stage	1.717	1.262–2.336	0.001	1.145	0.717–1.828	0.571

Variables in the equation.

### ERCC6L promotes the proliferation and migration of LUAD in vitro and in vivo

**Fig. 2:**
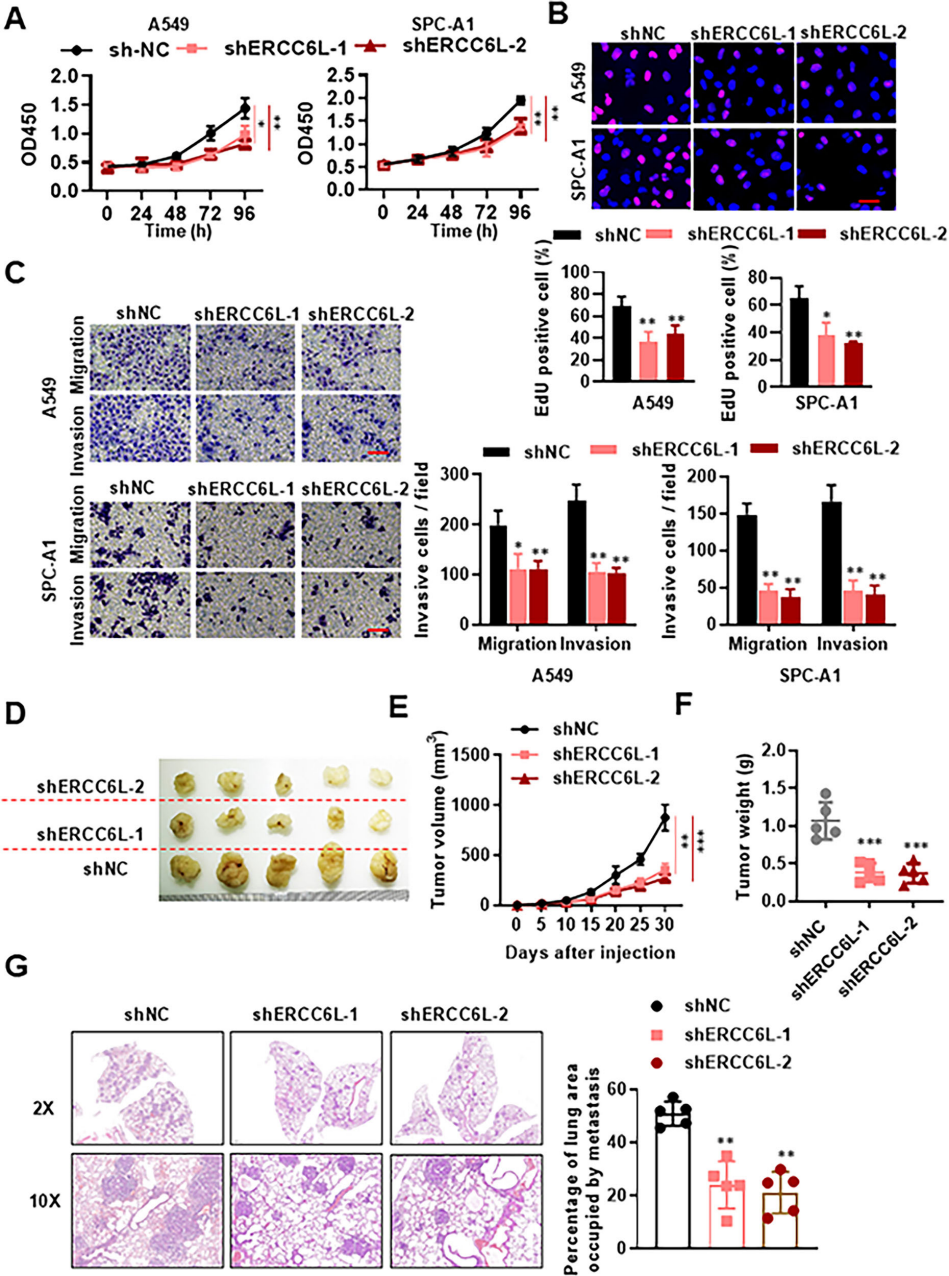
Knockdown of ERCC6L impedes the proliferation and migration of LUAD cells. **A**, **B** Impact of ERCC6L knockdown on A549 and SPC-A1 cell proliferation assessed by CCK-8 assay (**A**) and EdU assay (**B**). **C** Migration and invasion ability after ERCC6L knockdown in A549 and SPC-A1 cells using migration and transwell assays. **D**–**F** Representative images (**D**), tumor volume (**E**), and tumor weight (**F**) quantification over time. **G** HE staining and quantification of tumor occupancy in the lungs. Data are shown as the mean ± SEM. *p < 0.05, **p < 0.01, ***p < 0.001.

To delve into ERCC6L’s role in LUAD progression, lentivirus carrying short-hairpin RNA (shRNA) targeting ERCC6L (shERCC6L) was employed to infect A549 and SPC-A1 cells. Western blot confirmed efficient knockdown of ERCC6L in both A549 and SPC-A1 (Fig. S2A). Cell viability, assessed using the CCK-8 assay at various time points (0–4 days) post-infection, showed significantly decreased viability in shERCC6L-infected A549 and SPC-A1 cells compared to controls (Fig. 2A). Additionally, EdU staining indicated decreased proliferation capacity upon ERCC6L downregulation (Fig. 2B). Transwell assays revealed suppressed migration and invasion capabilities in LUAD cells upon ERCC6L knockdown (Fig. 2C).

To assess ERCC6L’s oncogenic role in vivo, xenograft tumor models were utilized. After 30 days, tumors were harvested, and their volume and weight were measured, showing significant suppression of tumor growth upon ERCC6L knockdown (Fig. 2D–F). Further investigation into ERCC6L knockdown’s influence on LUAD metastasis in vivo involved injecting A549 cells infected with shERCC6L-lentivirus into the tail vein of BABL/c nude mice. HE staining demonstrated inhibited colonization of LUAD cells in the lungs upon ERCC6L knockdown (Fig. 2G). In summary, our findings highlight the involvement of ERCC6L in promoting cell proliferation, migration, and invasion in LUAD.

### ERCC6L promotes stemness and chemoresistance in LUAD

**Fig. 3:**
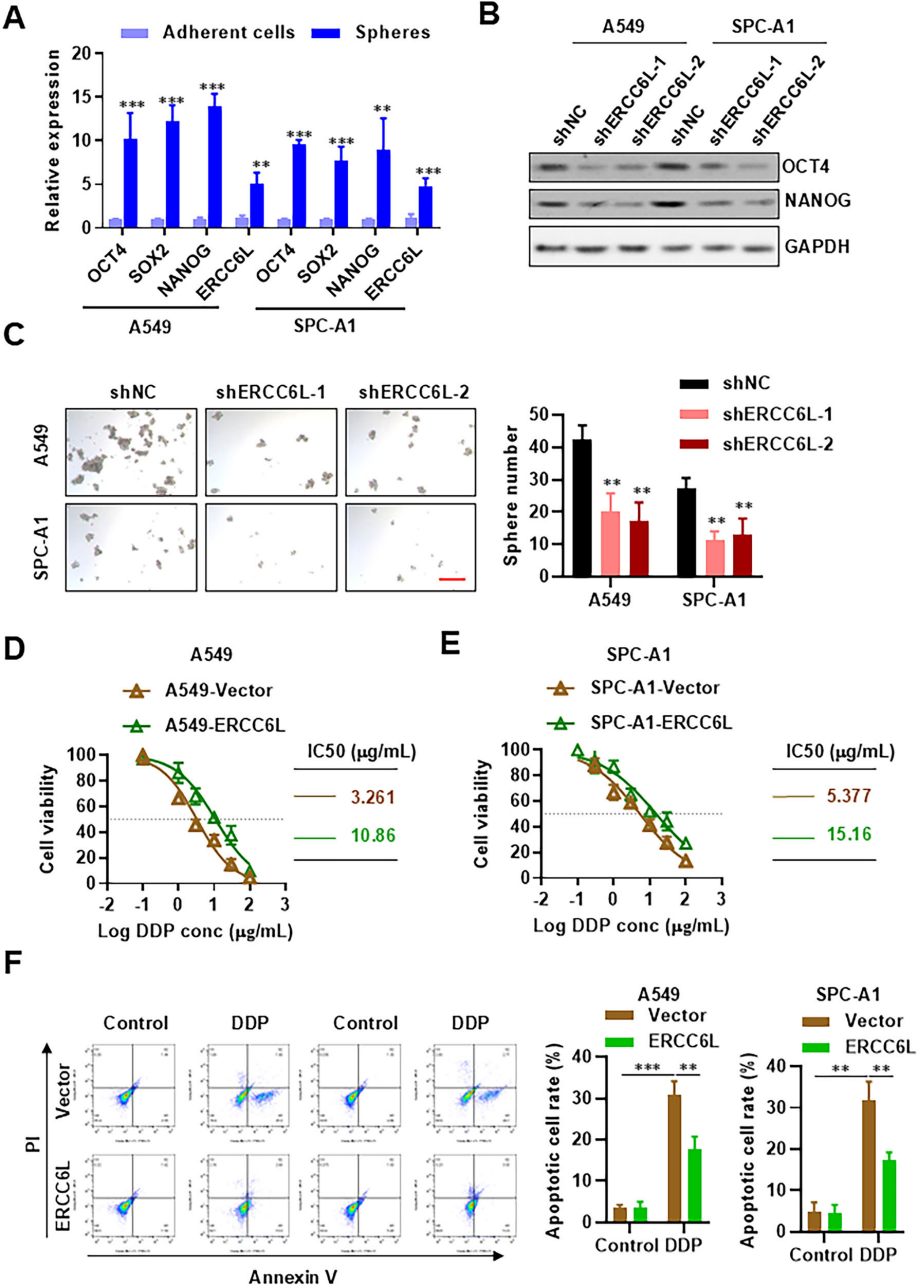
ERCC6L promotes stemness and cisplatin resistance in LUAD cells. **A** qRT-PCR analysis of OCT4, SOX2, NANOG, and ERCC6L mRNA expression in adherent cells or spheres of A549 and SPC-A1 cells. **B** Western blot analysis of OCT4, NANOG, and ERCC6L in ERCC6L-silenced stable A549 and SPC-A1 cells. **C** Sphere formation ability of ERCC6L-silenced A549 and SPC-A1 cells. **D**, **E** Effect of ERCC6L modulation on cisplatin sensitivity. **F** Apoptosis analysis of ERCC6L-overexpressing cells treated with cisplatin. Data are shown as the mean ± SEM. *p < 0.05, **p < 0.01, ***p < 0.001.

To investigate the influence of ERCC6L on the stemness of LUAD cancer cells, we initially assessed ERCC6L expression in CSC-like cells. Elevated ERCC6L expression was observed in CSC-like side populations of A549 and SPC-A1 separated via qPCR (Fig. 3A). Additionally, CSC-like cells capable of forming spheres exhibited increased ERCC6L and CSC markers (OCT4, SOX2, and NANOG)[29] expression (Fig. 3A). Further examination demonstrated that silencing ERCC6L significantly reduced the expression of OCT4, SOX2, and NANOG in A549 and SPC-A1 cells (Fig. 3B). Moreover, ERCC6L knockdown resulted in decreased levels of SOX2 and OCT4 in tumor tissues (Fig. S2C). Additionally, ERCC6L suppression in A549 and SPC-A1 cells markedly diminished their sphere formation capacities (Fig. 3C). Considering that CSCs are often implicated in chemotherapy resistance [30, 31], particularly to cisplatin, the primary chemotherapeutic agent for LUAD, we explored ERCC6L’s role in chemoresistance. Interestingly, ERCC6L overexpression substantially bolstered chemotherapy resistance and increased the IC50 value, while decreasing apoptosis in A549 and SPC-A1 cells treated with cisplatin (Fig. 3D–F). These findings suggest that ERCC6L maintains CSC-like characteristics in LUAD cells and facilitates chemoresistance in vitro.

### ERCC6L mediates glycolysis via upregulation of HIF-1α expression

**Fig. 4:**
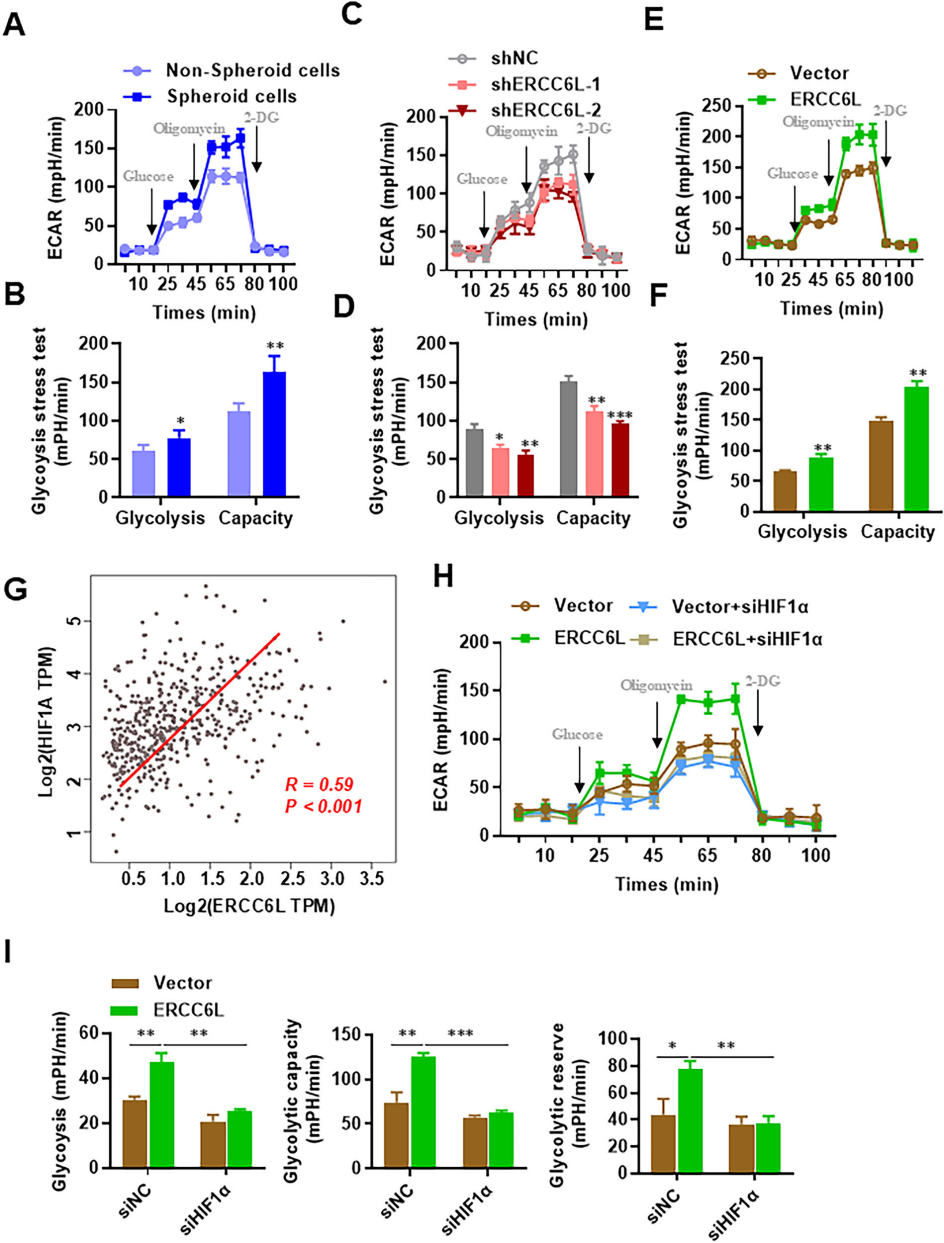
ERCC6L regulates LUAD glycolysis via HIF-1α. **A**, **B** Seahorse glycolytic rate assays in adherent cells or spheres of A549 cells. **C**, **D** Seahorse glycolytic rate assays in cells with ERCC6L knockdown or control cells. **E**, **F** Seahorse glycolytic rate assays in cells with ERCC6L overexpression or corresponding control cells. **G** Spearman correlation analysis of ERCC6L and HIF1A mRNA expression in the TCGA database. **H**, **I** ECAR measurements in ERCC6L-overexpressing A549 cells upon HIF-1α siRNA transfection. Data are shown as the mean ± SEM. *p < 0.05, **p < 0.01, ***p < 0.001.

Despite the well-established Warburg effect, the metabolic status of CSCs remains largely elusive. To gain insight into the metabolic state of CSCs, we examined extracellular acidification rates (ECAR) as a marker of glycolytic flux. ECAR analysis revealed an elevation in glycolysis and glycolytic capacity in spheroid cells compared to non-spheroid cells (Fig. 4A, B).

To assess ERCC6L’s impact on LUAD glycolytic flux, we transfected A549 cells with ERCC6L shRNAs or ERCC6L overexpression plasmids. As anticipated, ERCC6L knockdown led to glycolysis suppression in A549 cells, while ERCC6L overexpression exerted the opposite effect (Fig. 4C–F). HIF-1α, a key transcription factor regulating glycolysis[32], showed a positive correlation with ERCC6L expression in LUAD according to GEPIA database analysis (Fig. 4G). Additionally, HIF-1α knockdown in ERCC6L-overexpressing A549 cells not only reduced ECAR, as expected, but also nullified the increased glycolytic capacity mediated by ERCC6L overexpression (Fig. 4H, I). These results support the notion that ERCC6L promotes HIF-1α-dependent glycolysis signaling in LUAD cells.

### ERCC6L activates HIF-1α-dependent protumorigenic signals

**Fig. 5:**
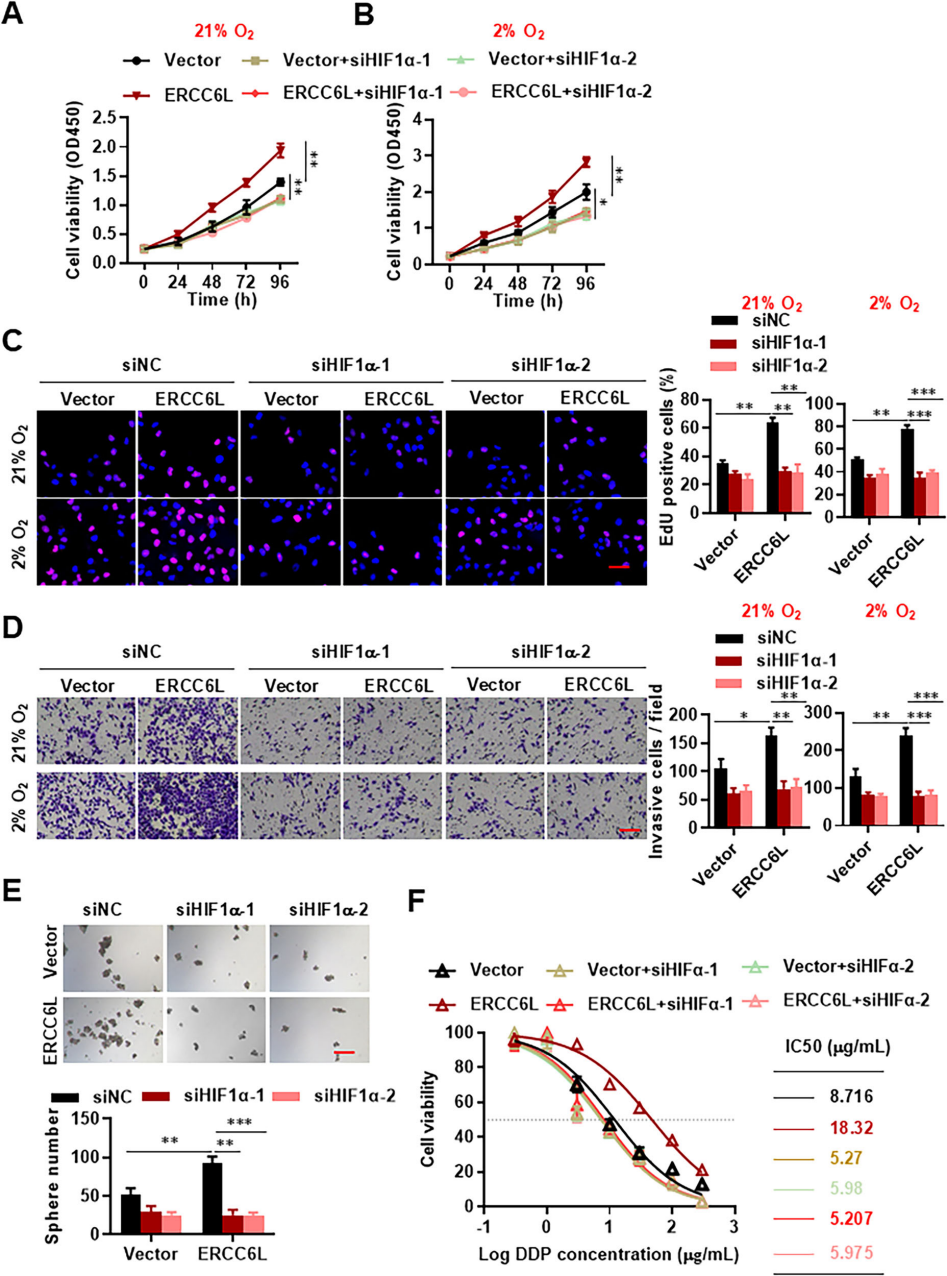
HIF-1α mediates ERCC6L-induced biological effects. **A**–**C** CCK8 assays (**A**, **B**) and EdU assay (**C**) in A549 cells with stable ERCC6L overexpression upon HIF-1α downregulation under normoxia and mild hypoxia. **D** Migration assay and quantification of cell migration. **E** Sphere formation ability of ERCC6L-overexpressed A549 cells with HIF-1α siRNA transfection. **F** CCK8 assay assessing cisplatin sensitivity in ERCC6L-overexpressing A549 cells with HIF-1α siRNA transfection. Data are shown as the mean ± SEM. *p < 0.05, **p < 0.01, ***p < 0.001.

To elucidate whether HIF1α participates in regulating the malignant features of lung adenocarcinoma mediated by ERCC6L, we silenced HIF1α in ERCC6L-overexpressing A549 cells. Western blot confirmed efficient knockdown of ERCC6L in both A549 and SPC-A1 (Fig. S2B). Results from the CCK8 and EdU assays indicate that ERCC6L overexpression significantly boosts A549 and SPC-A1 cell proliferation, and subsequent HIF1α knockdown completely reverses ERCC6L’s pro-proliferative effect under both normoxia (21% oxygen) and mild hypoxia (2% oxygen) conditions (Figs. 5A–C and S3A). Additionally, transwell assays were conducted to assess the impact of HIF1α on ERCC6L-promoted invasion in LUAD cells. The findings demonstrate that downregulation of HIF1α reverses ERCC6L’s promotion of cell invasion under both normoxia and mild hypoxia (Figs. 5D and S3B). Moreover, ERCC6L enhances sphere formation capacities in A549 and SPC-A1 cells, a phenomenon countered by HIF1α knockdown (Figs. 5E and S3C). Similarly, HIF1α knockdown reverses ERCC6L-induced chemoresistance (Fig. 5F). Moreover, we performed experiments in which HIF1α was overexpressed in an ERCC6L knockdown background. The results demonstrated that HIF1α overexpression significantly rescued the suppressed proliferation and migration of LUAD cells induced by ERCC6L silencing (Fig. S4A–C). Additionally, glycolysis stress tests confirmed that overexpression of HIF1α reversed the inhibition of glycolytic activity observed after ERCC6L knockdown (Fig. S4D). These findings strongly suggest that HIF-1α functions downstream of ERCC6L and is indispensable for ERCC6L’s facilitation of tumor progression.

### ERCC6L enhances HIF-1α transcriptional activity

**Fig. 6:**
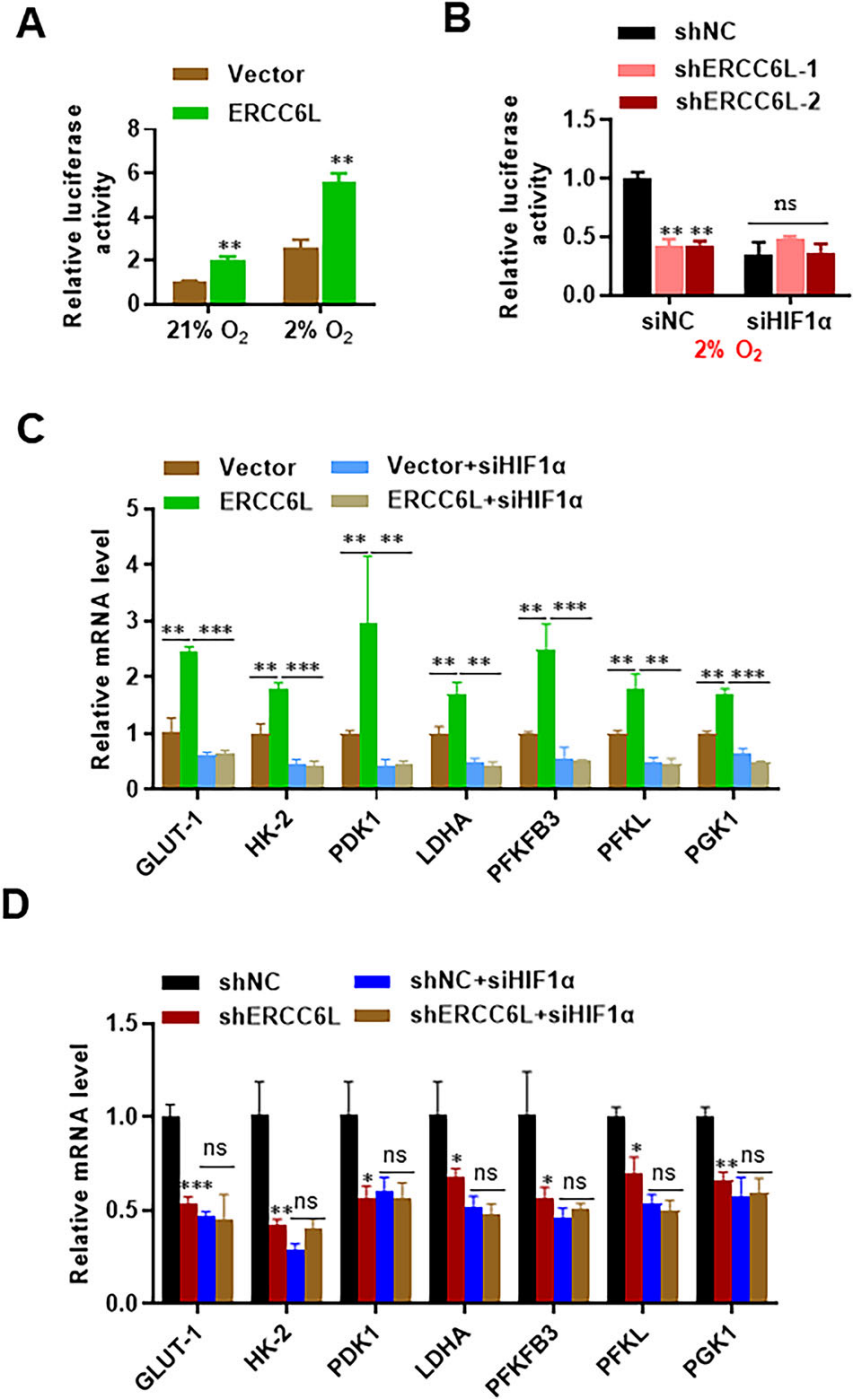
ERCC6L enhances HIF-1ɑ transcriptional activity. **A**, **B** Luciferase reporter assay measuring pGL3-HRE-luc activity of HIF-1α in ERCC6L-overexpressing A549 cells under 21% oxygen (**A**) or ERCC6L knockdown cells under 2% oxygen (**B**). **C** qRT-PCR analysis of glycolytic gene expression in ERCC6L-overexpressing A549 cells with or without HIF-1α siRNA transfection. **D** qRT-PCR analysis of glycolytic gene expression in ERCC6L-knockdown A549 cells with or without HIF-1α siRNA transfection. Data are shown as the mean ± SEM. *p < 0.05, **p < 0.01, ***p < 0.001.

We aimed to elucidate the underlying mechanism by which ERCC6L amplifies HIF-1α signaling. HIF-1α is a pivotal transcription factor regulating glycolysis by transcriptionally activating numerous target genes. Thus, we investigated whether ERCC6L modulates HIF-1α signaling by influencing its transcriptional activity. ERCC6L overexpression augmented HIF-1α‘s transcriptional activity, evidenced by increased HRE luciferase reporter activity under both normoxia and mild hypoxia (Fig. 6A). Hypoxia enhanced HRE luciferase activity, whereas ERCC6L knockdown mitigated hypoxia-induced HRE luciferase activity (Fig. 6B). Small interfering RNA-mediated inhibition of HIF-1α reduced HIF-1α expression and disrupted hypoxia-induced HRE luciferase activity, completely abolishing the effect of ERCC6L knockdown (Fig. 6B). These results suggest that ERCC6L promotes HRE luciferase activity under both normoxia and hypoxia in a HIF-1α-dependent manner.

Consistently, similar alterations in the mRNA levels of HIF-1α target glycolytic genes were observed upon ERCC6L overexpression or knockdown (Fig. 6C, D). Furthermore, HIF-1α knockdown diminished the expression of glycolytic genes targeted by HIF-1α, and ERCC6L lost its capacity to regulate these target genes’ expression in HIF-1α knockdown cells (Fig. 6D). These findings suggest that ERCC6L and HIF-1α collaborate to enhance the expression of HIF-1α targets via a shared pathway, supporting the concept that ERCC6L amplifies HIF-1α transcriptional activity.

### ERCC6L directly binds to VHL and prevents HIF-1α degradation

**Fig. 7:**
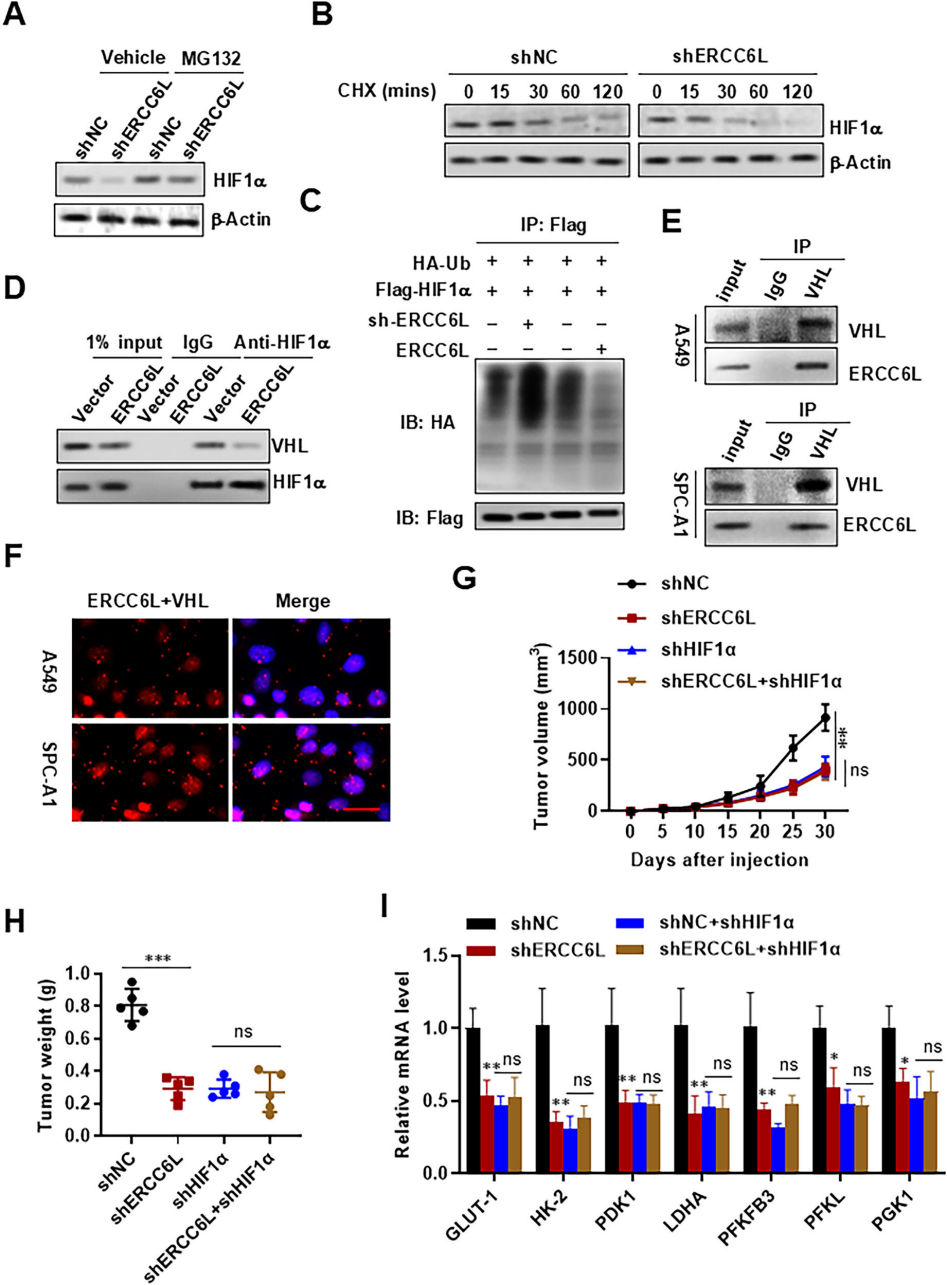
ERCC6L inhibits HIF-1α degradation by binding to VHL. **A** Western blot image of HIF-1α in ERCC6L knockdown cells with the proteasome inhibitor MG132 (40 μM). **B** Western blot images of HIF-1α in ERCC6L knockdown cells treated with 100 μg/ml CHX for varying time periods (0, 15, 30, 60, and 120 min). **C** Ub-HIF-1α levels determined by IP of anti-Flag followed by western blot with anti-HA after transfection of HIF-1α-Flag and Ub-HA in ERCC6L overexpression or knockdown 293 T cells with MG132 (20 μM). **D** The interaction between endogenous VHL and HIF-1α was determined by IP assay in ERCC6L overexpression cells. **E** The interaction between endogenous ERCC6L and VHL was determined by immunoprecipitation (IP) assay in A549 and SPC-A1 cells. **F** Proximity ligation assay was performed to examine the endogenous interaction between ERCC6L and VHL in A549 and SPC-A1 cells. Scale bar = 100 μm. **G**, **H** Tumor volumes (**G**) and tumor weight (**H**) in four different groups of A549 xenografts. **I** qRT-PCR analysis of glycolytic gene expression in A549 xenografts. Data are shown as the mean ± SEM. *p < 0.05, **p < 0.01, ***p < 0.001.

Our investigation delved into how ERCC6L impacts HIF-1α‘s transcriptional activity. As previously noted, immunohistochemistry revealed predominant cytoplasmic localization of ERCC6L (Fig. 1C), making direct interference with HIF-1α‘s nuclear binding to target genes improbable. Despite low HIF-1α expression under normoxia, detection of HIF-1α protein was feasible by loading 80 μg of total protein isolate from A549 cells. Under these conditions, ERCC6L overexpression consistently elevated basal HIF-1α levels (Fig. S5A). Additionally, ERCC6L knockdown diminished hypoxia-induced HIF-1α expression (Fig. S5B). Notably, this regulation did not extend to HIF-1α mRNA levels; neither ERCC6L overexpression nor knockdown notably influenced HIF-1α mRNA expression, regardless of oxygen concentration (Fig. S5C, D). Thus, we inferred that ERCC6L might modulate HIF-1α at the protein level. To test this hypothesis, ERCC6L knockdown cells underwent treatment with the proteasome inhibitor MG132. Remarkably, MG132 substantially increased HIF-1α expression, and under such conditions, ERCC6L knockdown-induced reduction in HIF-1α was nullified (Fig. 7A), indicating ERCC6L’s predominant role in inhibiting proteasome-mediated HIF-1α degradation. In accordance, ERCC6L knockdown significantly shortened HIF-1α half-life (Fig. 7B). Proteasomal degradation of target proteins primarily occurs via polyubiquitination. Our immunoprecipitation (IP) assay revealed polyubiquitinated HIF-1α, evident by higher molecular bands/smears. Notably, ERCC6L overexpression decreased polyubiquitinated HIF-1α, while ERCC6L knockdown increased it by approximately threefold (Fig. 7C). Hence, ERCC6L facilitates HIF-1α protein expression by impeding ubiquitin-mediated degradation. VHL serves as a crucial E3 ligase that directly binds with PHD-hydroxylated HIF-1α, instigating proteasome-mediated HIF-1α degradation [33]. We explored whether ERCC6L modulates HIF-1α stability by interfering with PHD/VHL-mediated HIF-1α degradation. Subsequent analysis confirmed endogenous interaction between ERCC6L and VHL. Notably, ERCC6L overexpression reduced the interaction between endogenous VHL and HIF-1α (Fig. 7D), indicating ERCC6L’s direct binding with VHL, rather than HIF-1α. We next tested the binding between ERCC6L and VHL. Our data showed that endogenous ERCC6L and VHL indeed interacted (Fig. 7E, F). These data provide evidence that ERCC6L directly binds to VHL and prevents HIF-1α degradation.

HIF-1α serves as a pivotal regulator of hypoxia adaptation. Consequently, we evaluated HIF-1α‘s contribution to ERCC6L-regulated tumor growth by genetically knocking down HIF-1α in A549 xenografts. Tumor growth significantly decreased by approximately threefold upon HIF-1α knockdown, with no further reduction observed with ERCC6L knockdown in HIF-1α-depleted xenografts (Fig. 7G, H), suggesting convergence of ERCC6L and HIF-1α mechanisms in this context. Additionally, HIF-1α knockdown reduced glycolytic gene levels of its targets, and ERCC6L silencing did not further diminish these target gene expressions in HIF-1α knockdown xenografts (Fig. 7I). These results underscore the indispensable role of HIF-1α signaling in ERCC6L-mediated tumor growth promotion.

## Discussion

Tumor heterogeneity and its implications for cancer prognosis and therapeutic response have recently garnered considerable attention [34, 35]. Tumors exhibit metabolic alterations compared to benign tissues, providing the energy and biomacromolecules necessary for malignant cell growth [15, 36]. HIF-1α signaling, as the master transcriptional factor of glycolytic metabolism, is frequently activated in human cancers [37]. While VHL mutation is crucial for constitutive HIF-1α activation in lung cancer [38], understanding how cancer cells activate HIF-1α in VHL wild-type cancers is a key research focus. Our findings present the initial evidence that ERCC6L, a previously identified oncogene, crucially regulates HIF-1α signaling under normoxia. Mechanistically, ERCC6L competes with HIF-1α for VHL binding, thereby obstructing VHL-mediated HIF-1α degradation. Notably, increased ERCC6L expression in LUAD correlates with elevated HIF-1α levels, suggesting that upregulation of oncogenic ERCC6L may contribute to HIF-1α activation in vivo.

While excision repair cross-complementing group 6 (ERCC6) has been linked to lung cancer risk, its specific roles in LUAD progression are poorly understood. To date, the precise mechanism by which ERCC6L regulates tumor cell development and progression in LUAD remains unclear. Our study reveals that ERCC6L enhances proliferation and migration and promotes cancer stemness in LUAD cells. Growing evidence suggests that cancer stem cells (CSCs) confer resistance to conventional therapies due to their stemness properties [39]. Here, we demonstrate that ERCC6L plays a critical role in promoting lung cancer stemness by stabilizing HIF-1α expression. This conclusion is supported by the observation that ectopic expression of ERCC6L significantly upregulates NANOG, Oct4, and Sox2 expression (Fig. S6A), increases the population of CD133^+^ cells (Fig. S6B), all of which can be rescued by concurrent silencing of HIF-1α. These results suggest that ERCC6L is involved in HIF-1α stabilization in LUAD cells, contributing to the malignant progression of these tumor cells.

Recent studies have highlighted the diversity of tumor metabolism in vivo, emphasizing the significant influence of the microenvironment on this aspect [40, 41]. The metabolic profile of cancer stem cells (CSCs) has been extensively investigated, as it potentially maintains CSC cellular states crucial for tumor initiation and metastasis. However, CSC metabolic phenotypes vary among different tumor types [42, 43]. Our findings in this manuscript reveal that CSCs derived from the human lung cancer line A549 exhibit characteristics of aerobic glycolysis metabolism. HIF-1α, a key transcription factor involved in cellular oxygen sensing and hypoxic adaptation, regulates the expression of glycolytic genes essential for various metabolic processes such as glucose uptake, glycolysis, and lactate excretion [44]. Previous research has shown that post-translational modifications of HIF-1α or VHL affect their binding capacities, thereby influencing HIF-1α protein stability. For instance, modifications such as deSUMOylation by SENP1, methylation by SET7/9 [45], or phosphorylation by p38 can alter the interaction between HIF-1α and VHL, leading to HIF-1α accumulation and activation. Conversely, acetylation of HIF-1α facilitates its binding with VHL, promoting HIF-1α degradation [46]. Additionally, VHL mutations at the HIF-1α-binding site underscore the crucial role of the VHL-HIF-1α interaction in VHL-mediated inhibition of HIF-1α [47]. Given the significance of the VHL-HIF-1α interaction in determining HIF-1α abundance, efforts have been made to develop small-molecule drugs targeting this interaction for the treatment of conditions like chronic anemia and ischemia [48]. Consistent with these observations, our study demonstrates that ERCC6L disrupts the HIF-1α-VHL interaction by competitively binding with VHL, leading to robust activation of HIF-1α in cancer cells. This represents the first evidence of ERCC6L’s role in activating HIF-1α activity, suggesting a novel avenue for anticancer treatment development.

In conclusion, our study revealed that ERCC6L was upregulated in LUAD spheroid cells with enhanced stemness compared to parental cells, correlating with poor prognosis in LUAD patients. Functionally, ERCC6L promoted LUAD cell stem-like characteristics by regulating tumor glycolysis via the VHL/HIF-1α pathways (Fig. S7). These findings imply that targeting ERCC6L may offer a promising therapeutic approach for LUAD treatment through modulation of the HIF-1α pathway.

## Supplementary information

Supplementary figure

Original Western blots

## Data Availability

The data analyzed during this study are included in this published article and the supplemental data files.
