# Supplementary material for: ERCC6L-mediated stabilization of HIF-1α enhances glycolysis and stemness properties of lung adenocarcinoma cells
Source: Cell Death Dis. 2025 Jul 21;16(1):541. doi: 10.1038/s41419-025-07879-4 (PMC12280123; doi:10.1038/s41419-025-07879-4)
Supplement: Supplementary file 1 — Supplementary figure [file 41419_2025_7879_MOESM1_ESM.pdf]

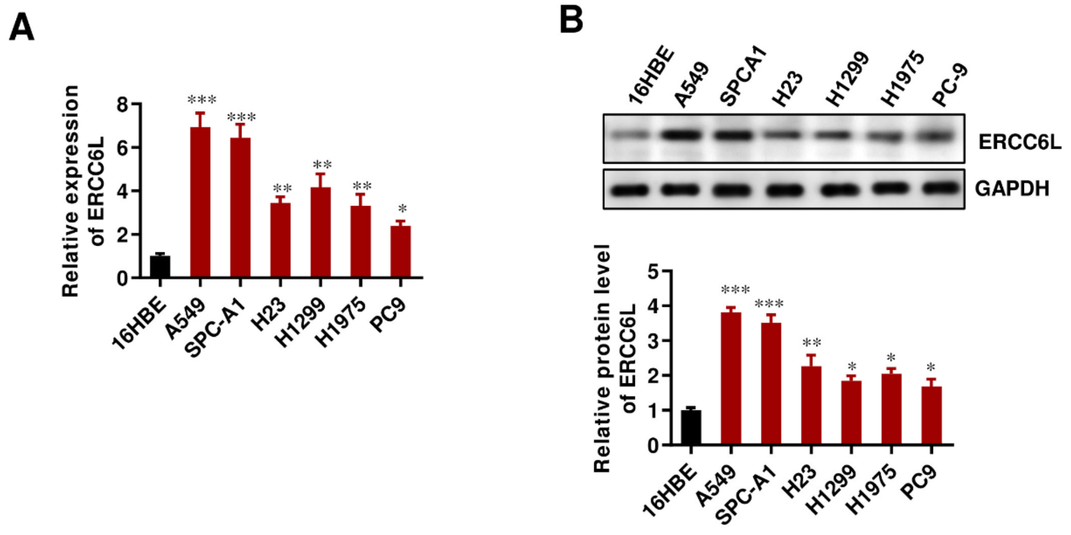

Figure S1. A-B. Relative ERCC6L mRNA (A) and protein (B) expression in LUAD cell lines compared to normal human bronchial epithelial cells. Data are shown as the mean ± SEM. *p<0.05, **p< 0.01, ***p< 0.001.

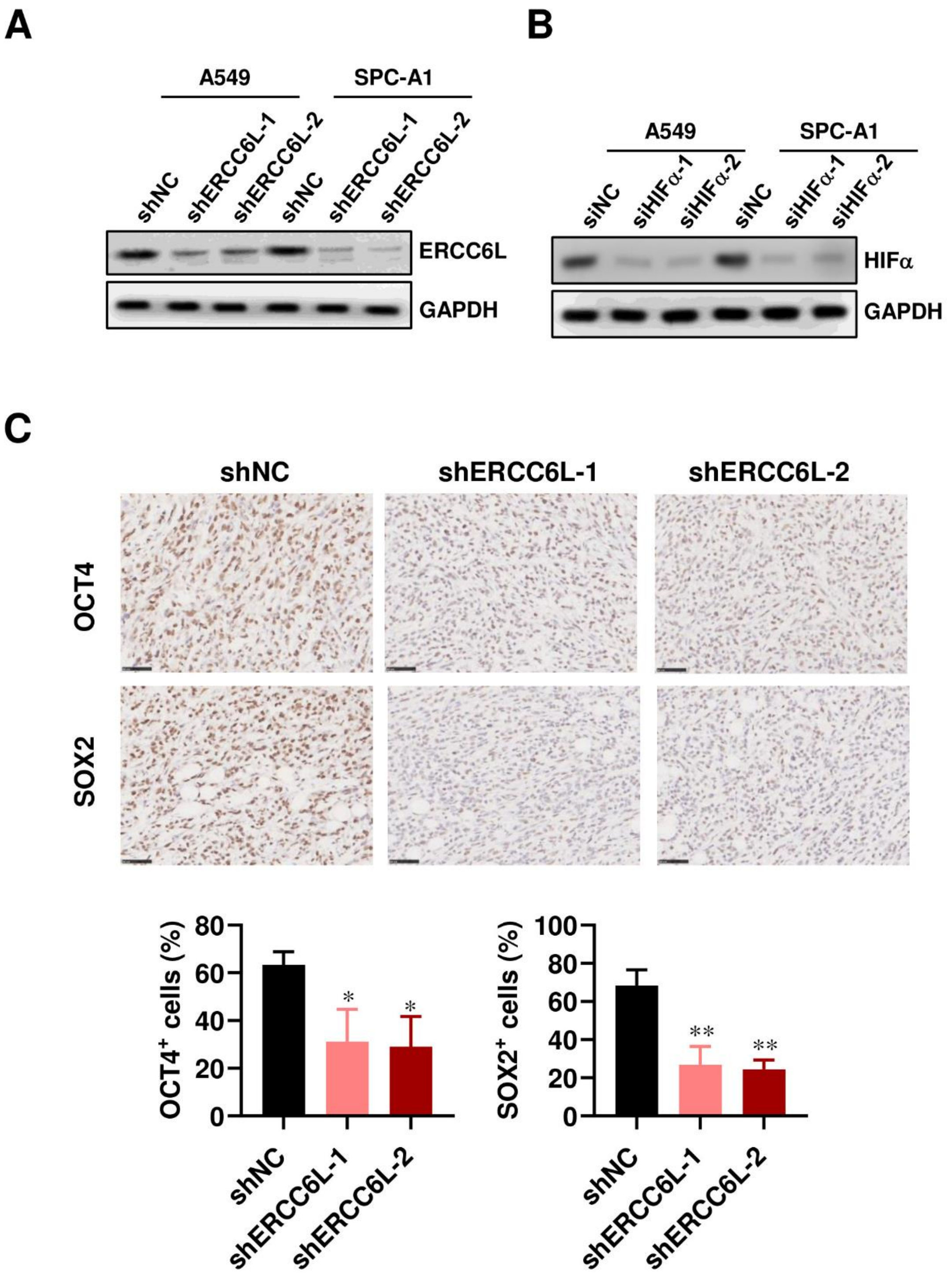

Figure S2. A. Western blot verification of knockdown efficiency of specific shRNA for ERCC6L in A549 and SPC-A1 cells. B. Western blot verification of knockdown efficiency of specific siRNA for HIF1α in A549 and SPC-A1 cells. **C.** Immunohistochemical staining for SOX2 and OCT4 in the indicated tumor xenografts (Scale bars: 50 μm), with quantification of positively stained cells. Data are shown as the mean ± SEM. $^*p<0.05$, $^{**}p< 0.01$, $^{***}p< 0.001$.

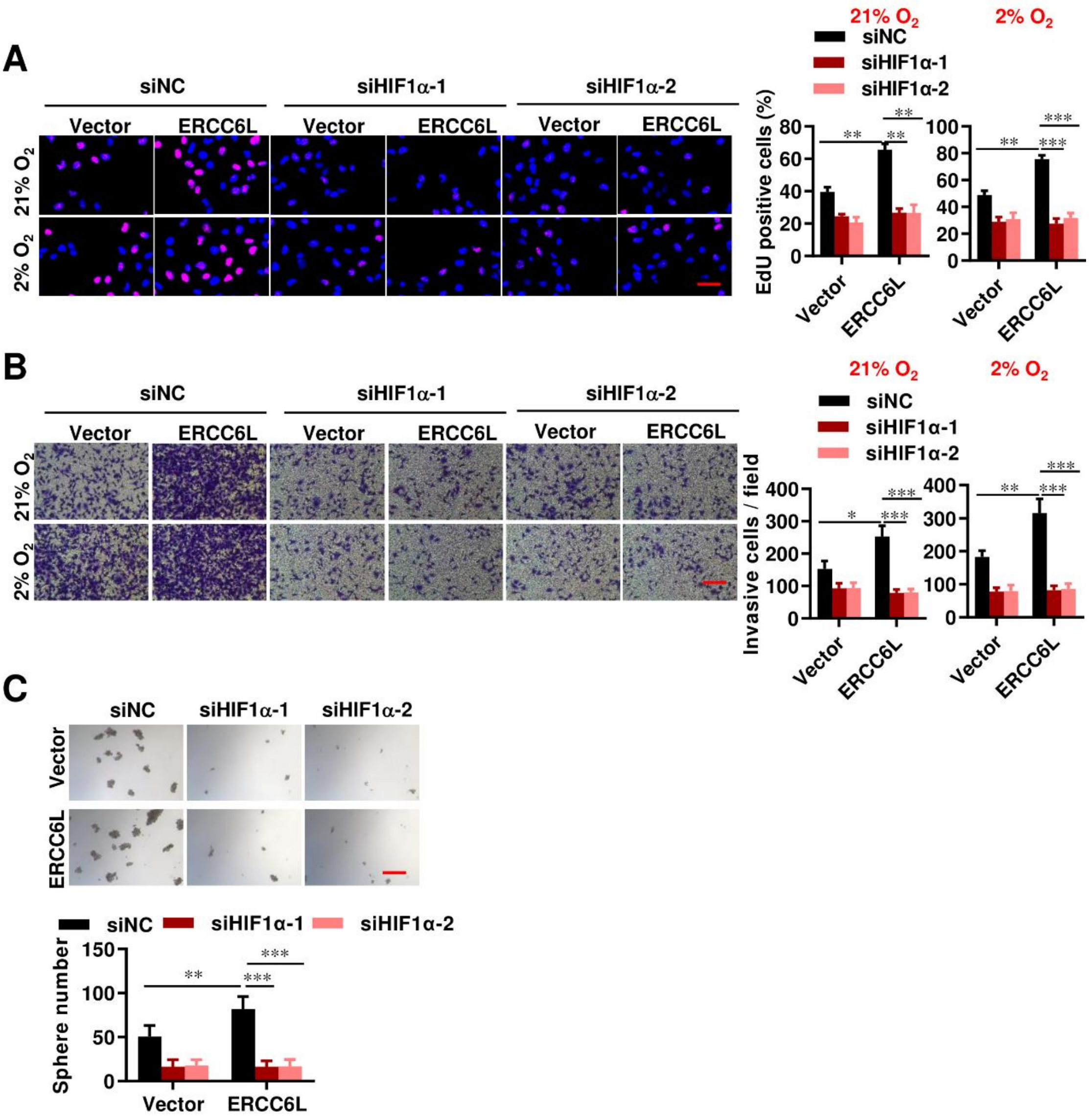

Figure S3. HIF-1α mediates ERCC6L-induced biological effects. A. EdU assay in SPC-A1 cells with stable ERCC6L overexpression upon HIF-1α downregulation under normoxia and mild hypoxia. B. Migration assay and quantification of cell migration. C. Sphere formation ability of ERCC6L-overexpressed SPC-A1 cells with HIF-1α siRNA transfection. Data are shown as the mean ± SEM. $*p<0.05$, $**p< 0.01$, $***p< 0.001$.

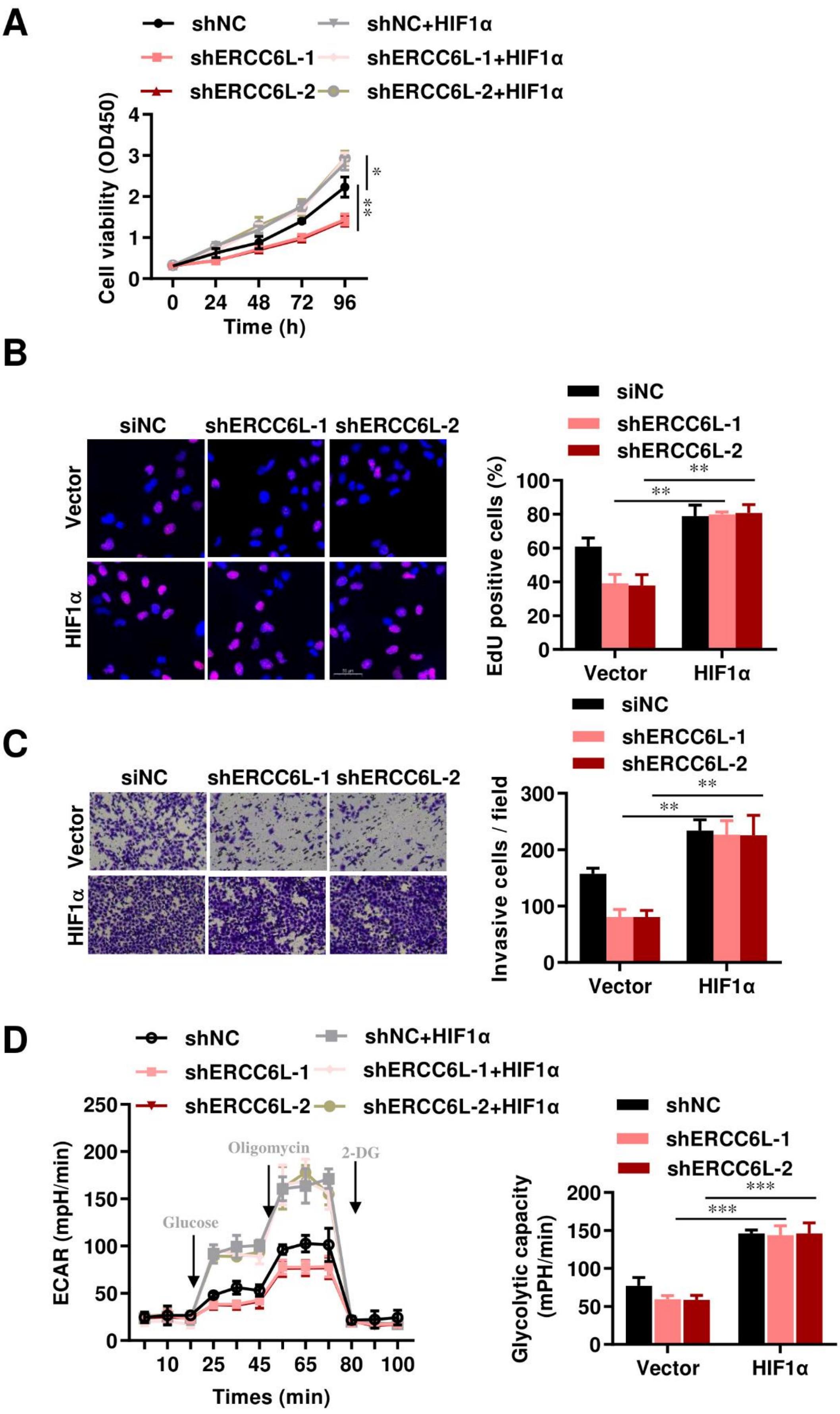

A
shNC
shNC+HIF1α
shERCC6L-1
shERCC6L-1+HIF1α
shERCC6L-2
shERCC6L-2+HIF1α
Cell viability (OD450)
Time (h)
B
siNC
shERCC6L-1
shERCC6L-2
Vector
HIF1α
EdU positive cells (%)
C
Invasive cells / field
D
ECAR (mpH/min)
Glucose
Oligomycin
2-DG
Times (min)
Glycolytic capacity (mPH/min)

Figure S4. HIF-1α mediates ERCC6L-induced biological effects. A-B. CCK8 assay (A) and EdU assay (B) assessing the proliferation of A549 cells with stable ERCC6L knockdown and HIF-1α overexpression under mild hypoxia. C. Transwell invasion assay with quantification, measuring the invasive capacity of A549 cells. D. Extracellular acidification rate measurements in ERCC6L-knockdown A549 cells upon HIF-1α overexpression. Data are shown as the mean ± SEM. *$p<0.05$, **$p< 0.01$, ***$p< 0.001$.

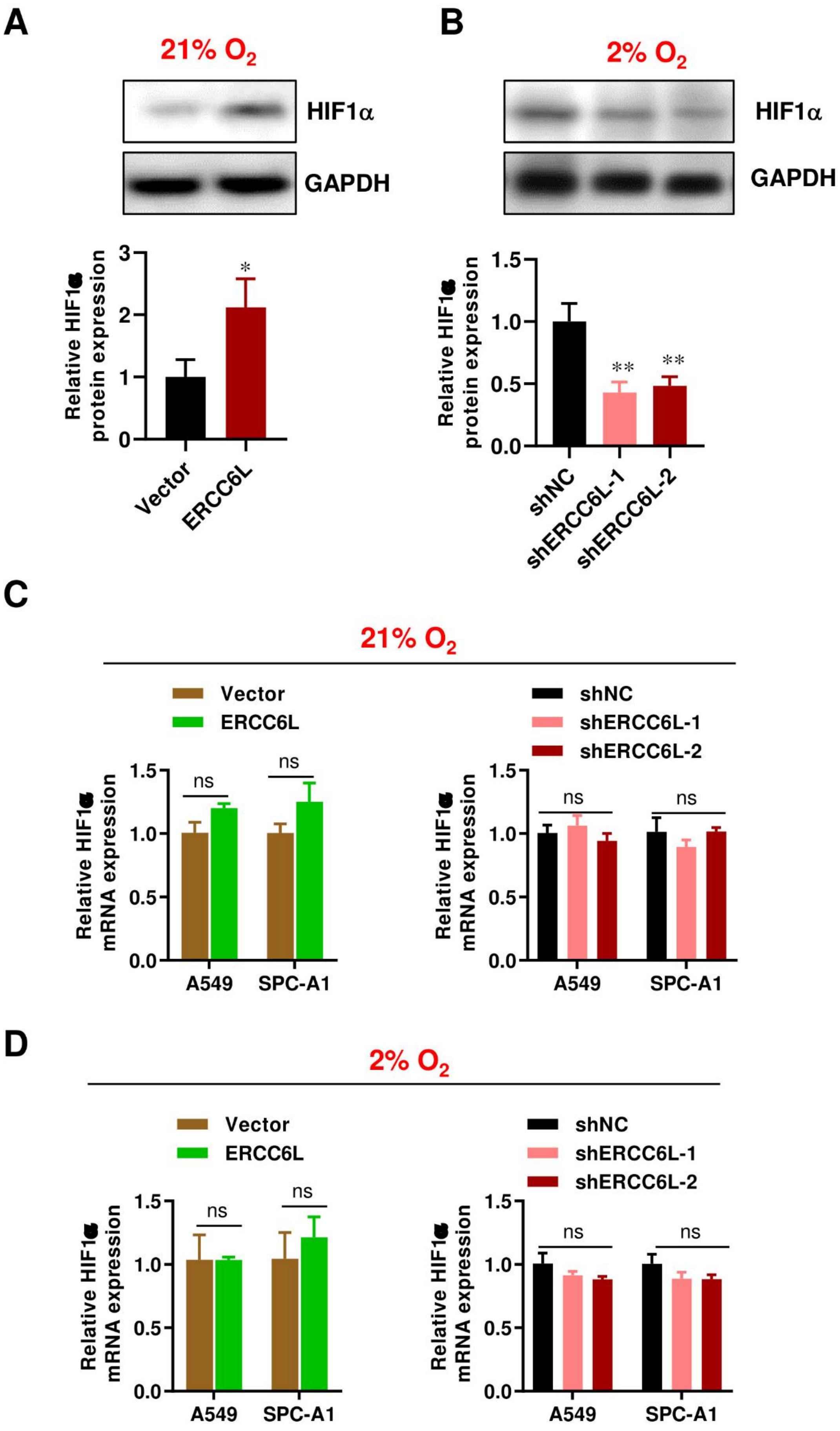

A
21% O_2
HIF1α
GAPDH
Relative HIF1α protein expression
Vector
ERCC6L
B
2% O_2
HIF1α
GAPDH
Relative HIF1α protein expression
shNC
shERCC6L-1
shERCC6L-2
C
21% O_2
Vector
ERCC6L
shNC
shERCC6L-1
shERCC6L-2
Relative HIF1α mRNA expression
ns
A549
SPC-A1
D
2% O_2
Vector
ERCC6L
shNC
shERCC6L-1
shERCC6L-2
Relative HIF1α mRNA expression
ns
A549
SPC-A1

Figure S5. ERCC6L stabilizes HIF-1α protein. A-B. Western blot images of HIF-1α in ERCC6L overexpression (A) or knockdown (B) A549 cells under 21% (A) or 2% oxygen (B). C-D. mRNA levels of HIF-1α under different treatments. Data are shown as the mean ± SEM. *$p<0.05$, **$p<0.01$, ***$p<0.001$.

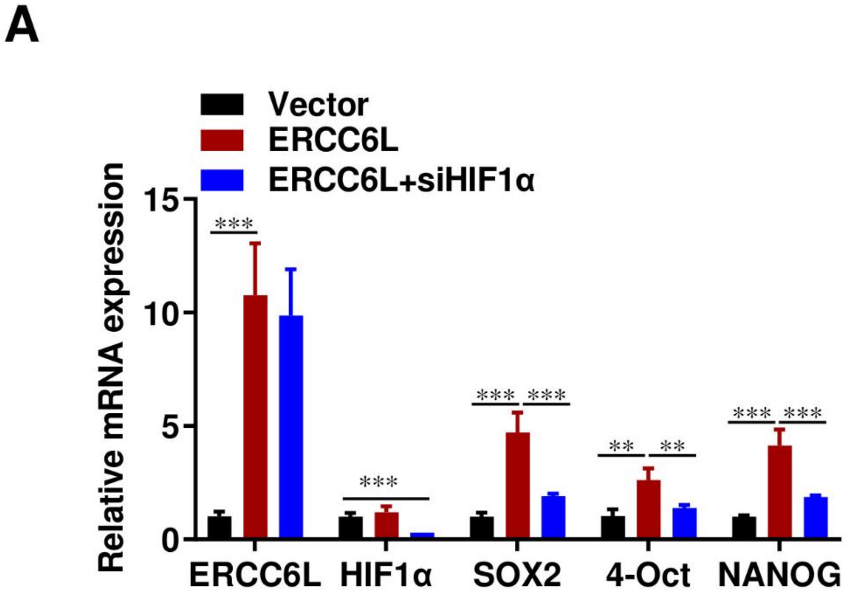

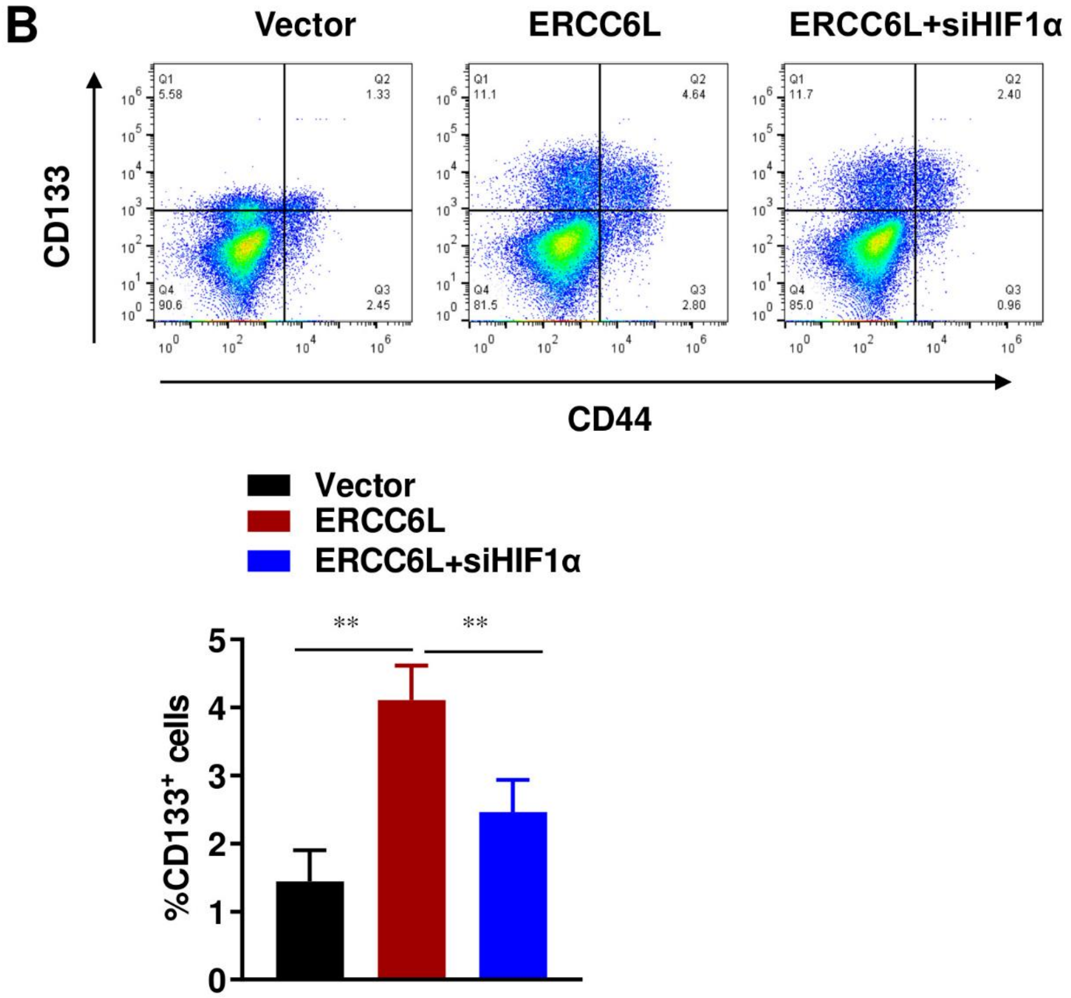

Figure S6. A. A549 cells stably expressing ERCC6L and either transfected with siHIF1a or siNC were subjected to qPCR analyses. B. FACS analyses for CD133-stained cells. Respective images

and quantitation were shown. Data are shown as the mean ± SEM. **$p < 0.01$, ***$p < 0.001$.

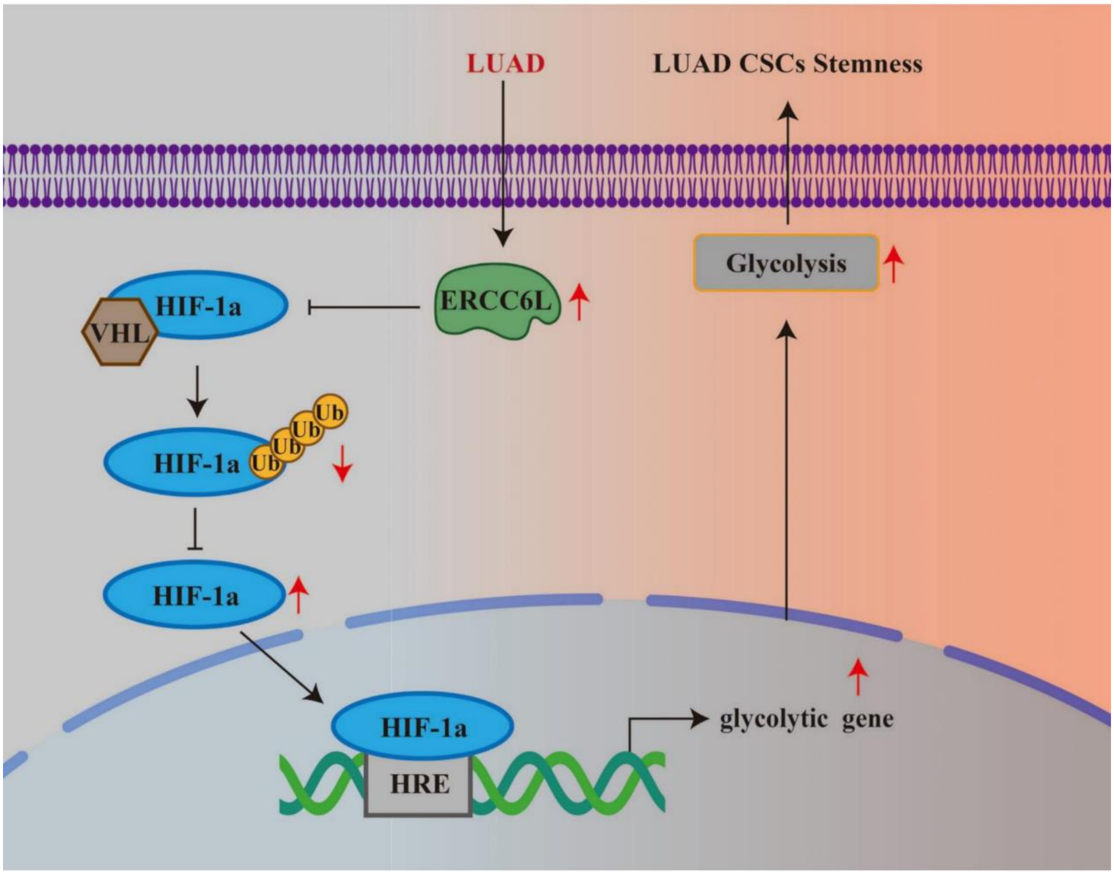

Figure S7. Schematic diagram of the ERCC6L/HIF-1α axis in the malignant properties of lung adenocarcinoma cells.
