# Supplementary figures and images for: ERCC6L-mediated stabilization of HIF-1α enhances glycolysis and stemness properties of lung adenocarcinoma cells
Source: Cell Death Dis. 2025 Jul 21;16(1):541. doi: 10.1038/s41419-025-07879-4 (PMC12280123; doi:10.1038/s41419-025-07879-4)

Figure 3

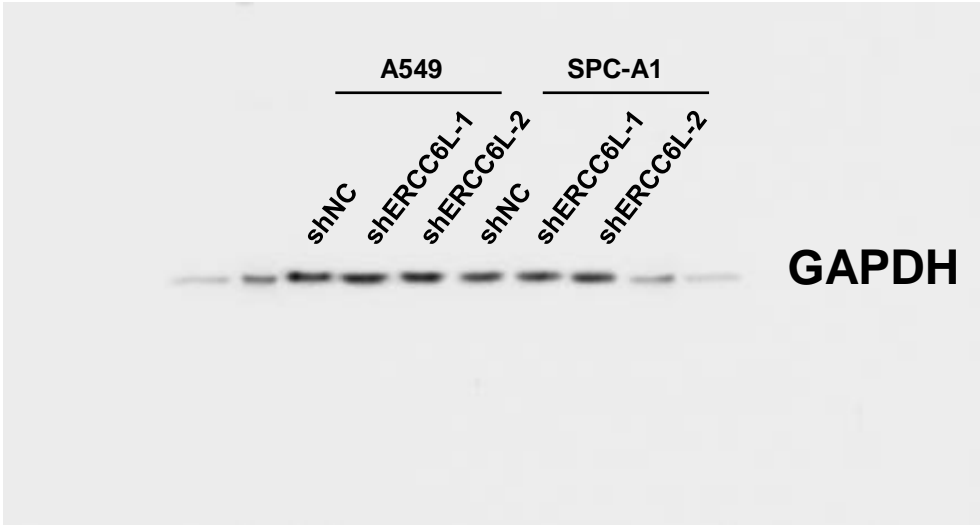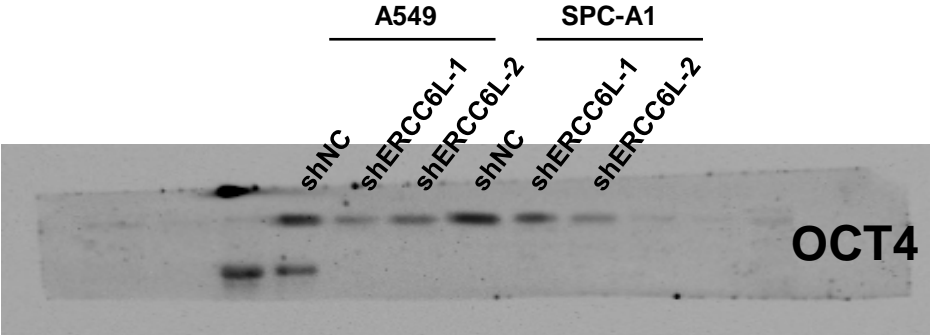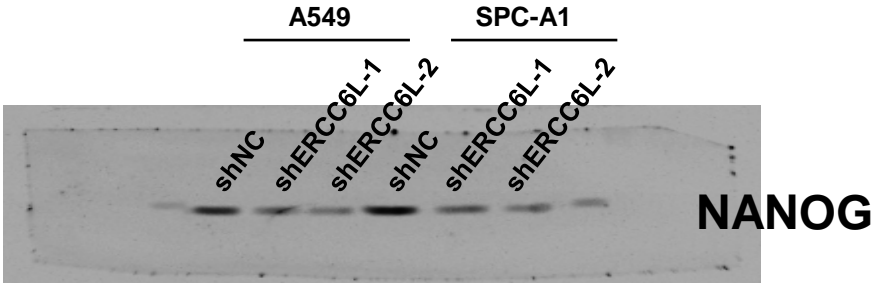

Figure 7

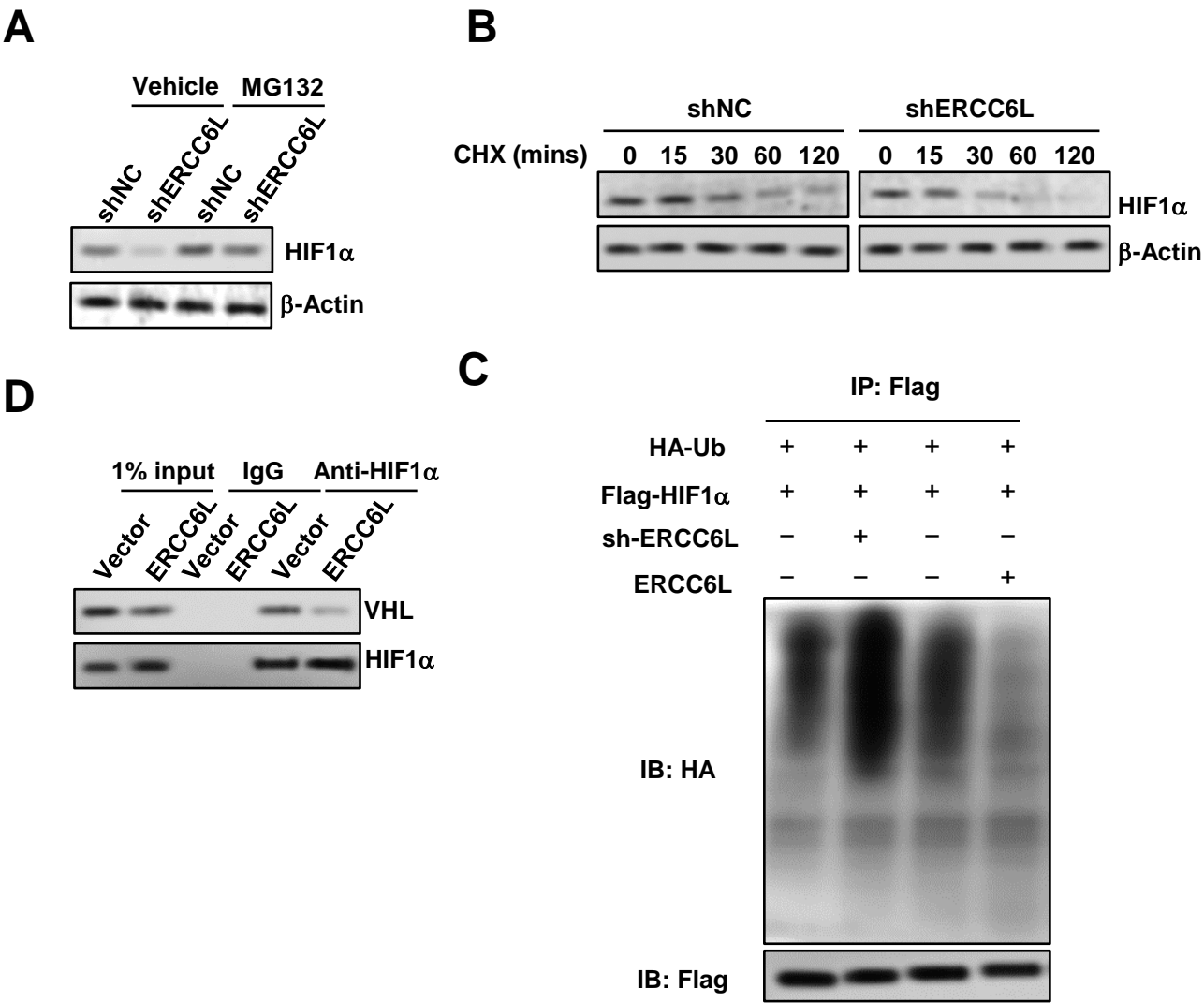

Figure 7

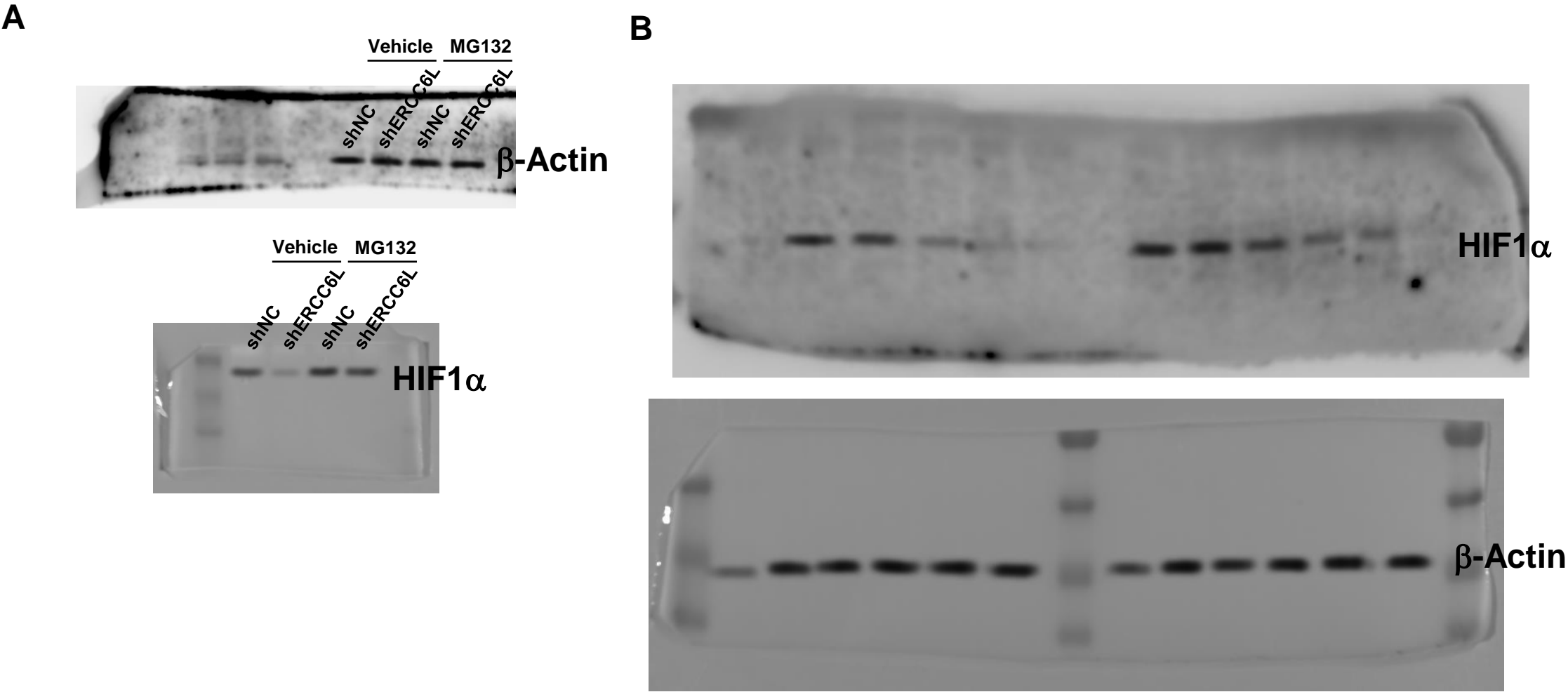

Figure 7C

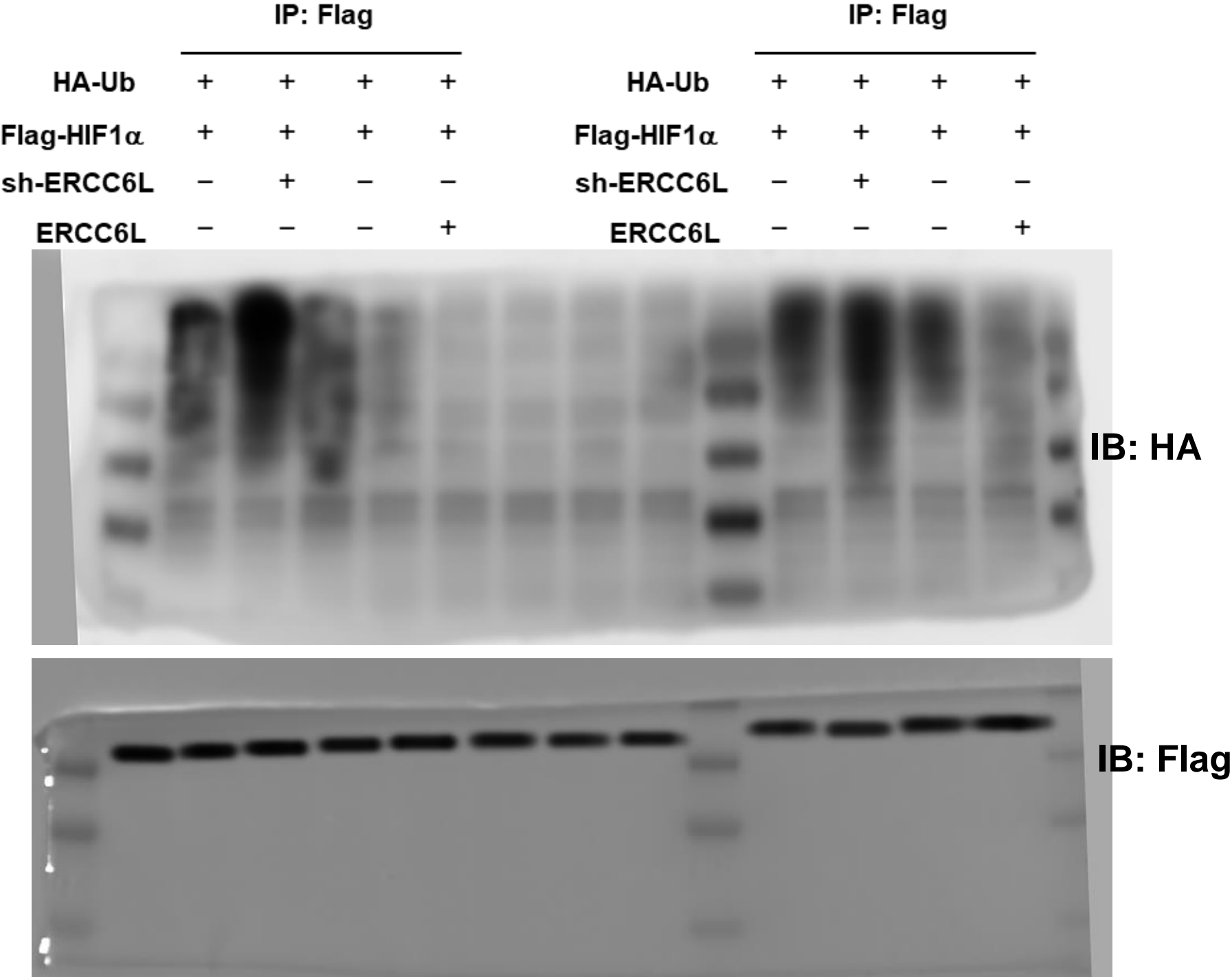

Figure 7D

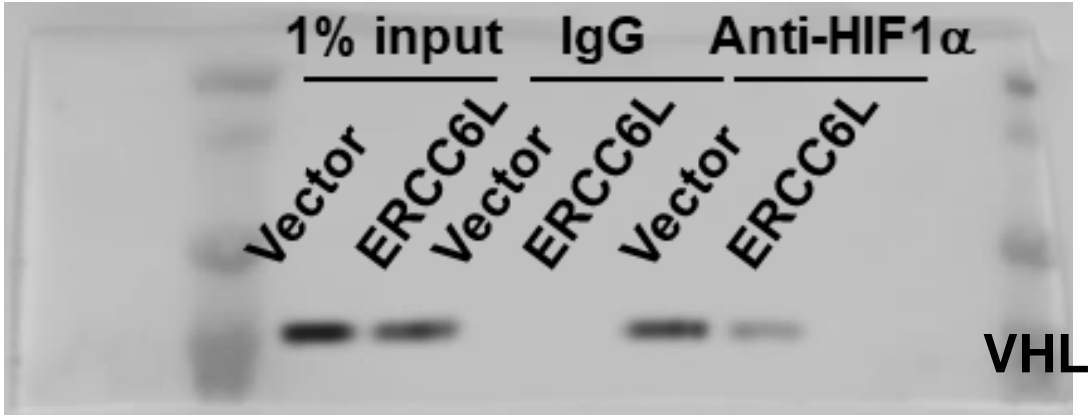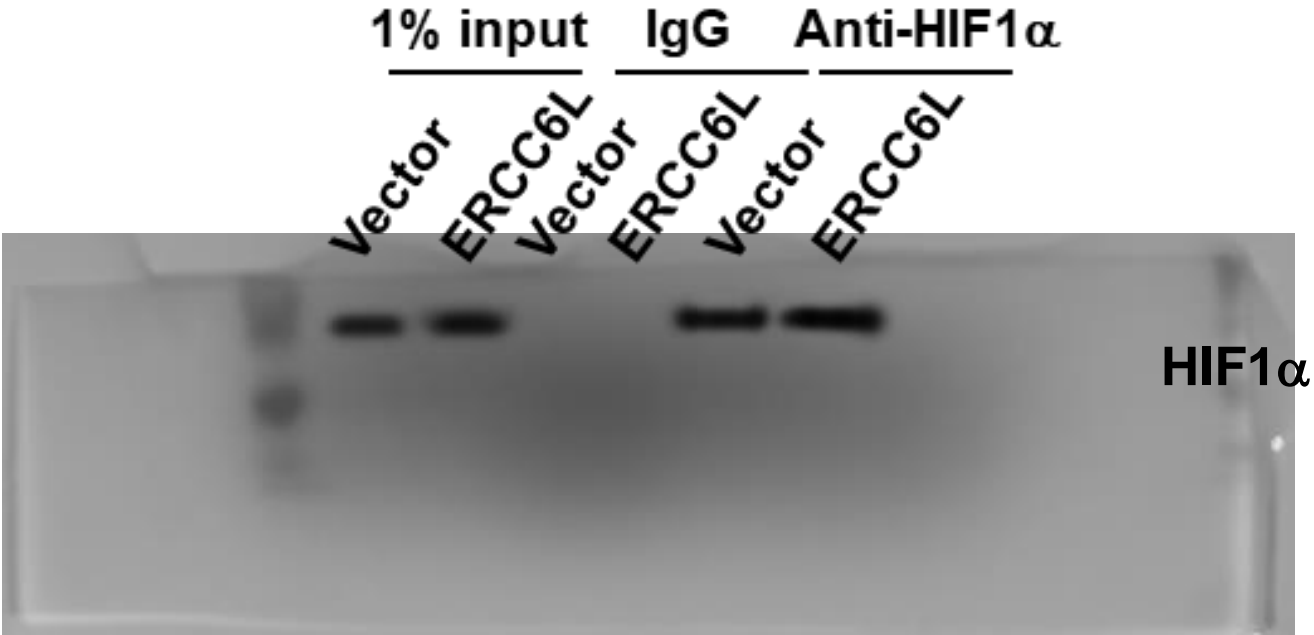

Figure S1B

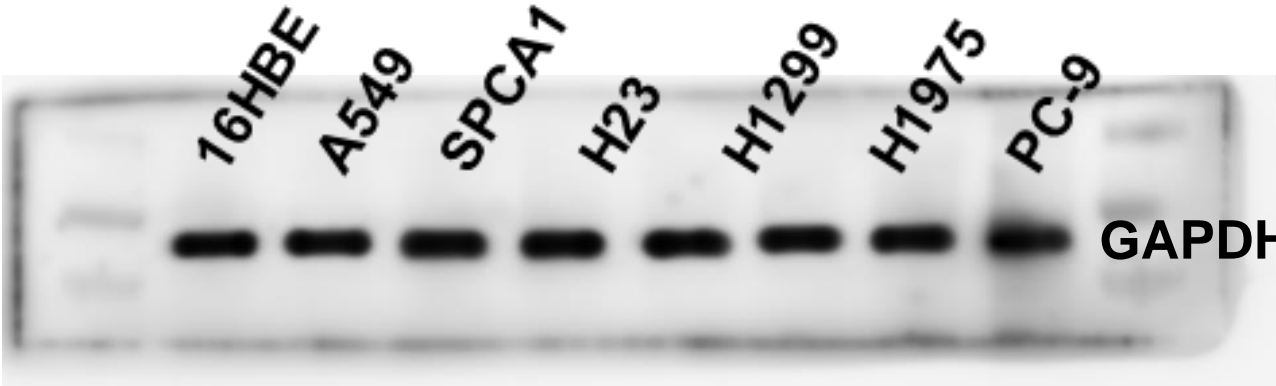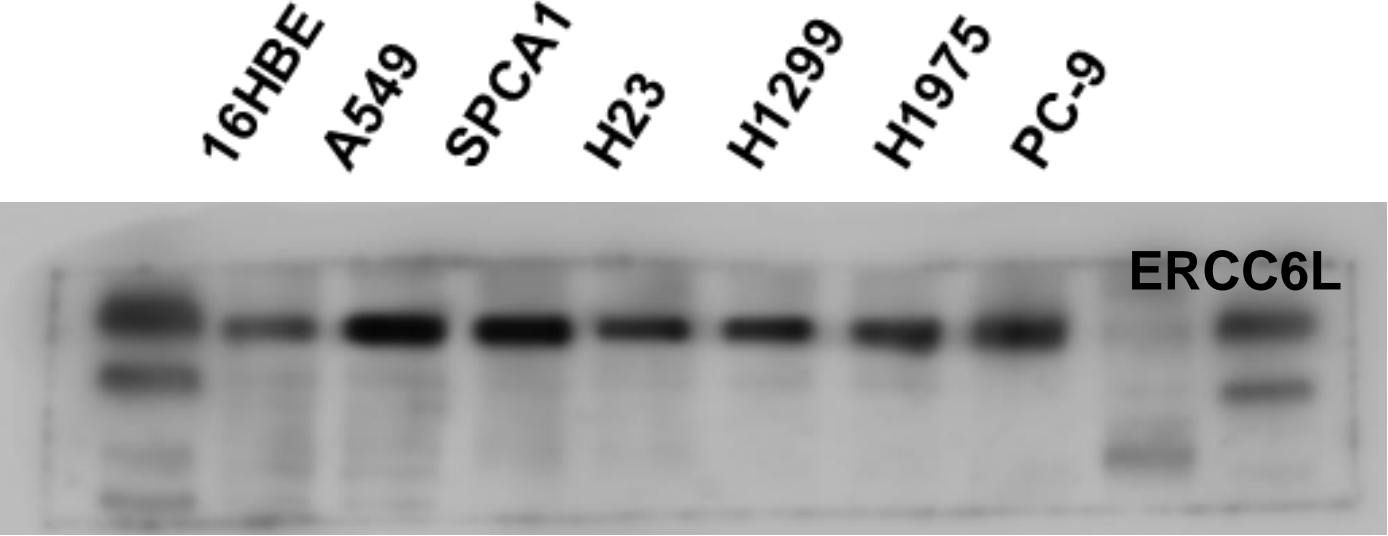

Figure S2

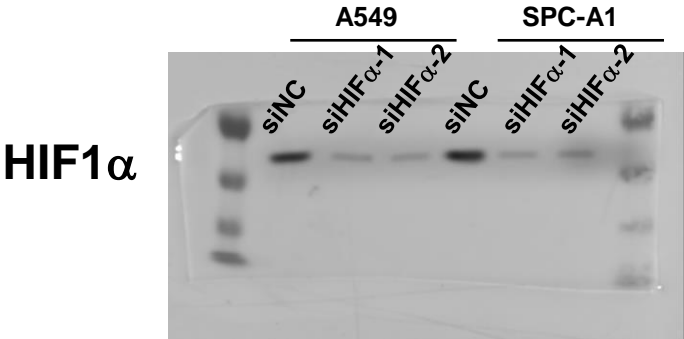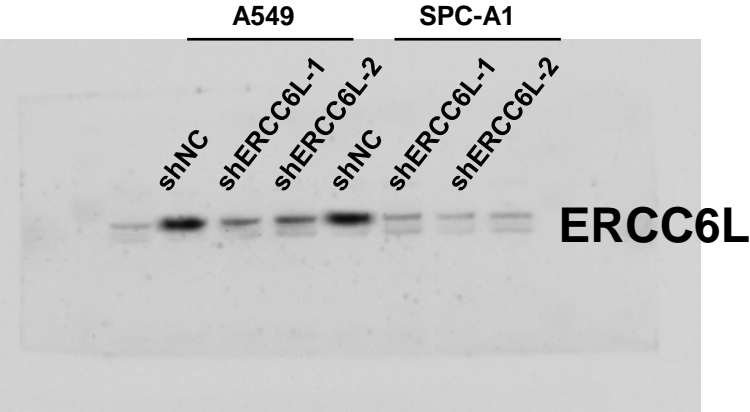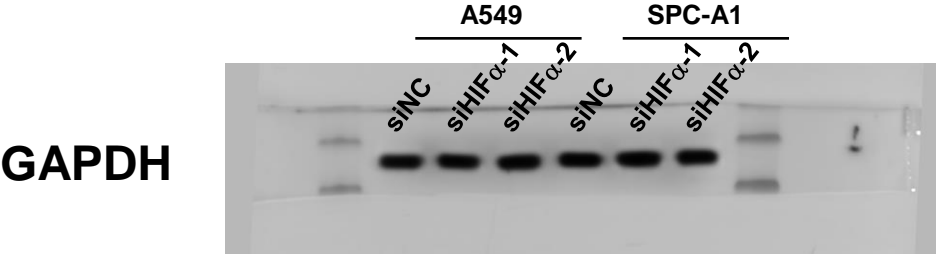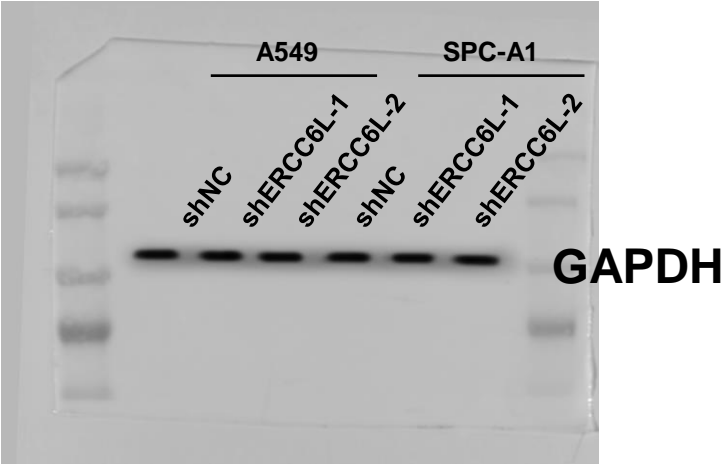

Supplement: Supplementary file 2 — Original Western blots [file 41419_2025_7879_MOESM2_ESM.pdf]
